# BTB-BACK Domain Protein POB1 Suppresses Immune Cell Death by Targeting Ubiquitin E3 ligase PUB17 for Degradation

**DOI:** 10.1371/journal.pgen.1006540

**Published:** 2017-01-05

**Authors:** Beatriz Orosa, Qin He, Joelle Mesmar, Eleanor M. Gilroy, Hazel McLellan, Chengwei Yang, Adam Craig, Mark Bailey, Cunjin Zhang, Jonathan David Moore, Petra C. Boevink, Zhendong Tian, Paul R. J. Birch, Ari Sadanandom

**Affiliations:** 1 School of Biological and Biomedical Sciences, Durham University, United Kingdom; 2 Division of Plant Sciences, University of Dundee (at JHI), Invergowrie, Dundee, United Kingdom; 3 Key Laboratory of Horticultural Plant Biology (HAU), Ministry of Education, Huazhong Agricultural University, Wuhan, Hubei, China; 4 Cell and Molecular Sciences, James Hutton Institute, Invergowrie, Dundee, United Kingdom; 5 Warwick Systems Biology Centre, University of Warwick, United Kingdom; University of California Davis, UNITED STATES

## Abstract

Hypersensitive response programmed cell death (HR-PCD) is a critical feature in plant immunity required for pathogen restriction and prevention of disease development. The precise control of this process is paramount to cell survival and an effective immune response. The discovery of new components that function to suppress HR-PCD will be instrumental in understanding the regulation of this fundamental mechanism. Here we report the identification and characterisation of a BTB domain E3 ligase protein, POB1, that functions to suppress HR-PCD triggered by evolutionarily diverse pathogens. *Nicotiana benthamiana* and tobacco plants with reduced POB1 activity show accelerated HR-PCD whilst those with increased POB1 levels show attenuated HR-PCD. We demonstrate that POB1 dimerization and nuclear localization are vital for its function in HR-PCD suppression. Using protein-protein interaction assays, we identify the Plant U-Box E3 ligase PUB17, a well established positive regulator of plant innate immunity, as a target for POB1-mediated proteasomal degradation. Using confocal imaging and *in planta* immunoprecipitation assays we show that POB1 interacts with PUB17 in the nucleus and stimulates its degradation. Mutated versions of POB1 that show reduced interaction with PUB17 fail to suppress HR-PCD, indicating that POB1-mediated degradation of PUB17 U-box E3 ligase is an important step for negative regulation of specific immune pathways in plants. Our data reveals a new mechanism for BTB domain proteins in suppressing HR-PCD in plant innate immune responses.

## Author Summary

Control over PCD in plants, like in animals, is central to determining susceptibility or resistance to disease. Yet there is a real paucity in understanding of the biochemical processes that are crucial in determining how PCD is co-ordinated during plant immune responses. Here we demonstrate that a BTB domain protein, POB1, is a conserved novel negative regulator of plant immune responses triggered by evolutionarily diverse pathogens. BTB domain proteins have been shown to associate with Cullin-3 proteins to form ubiquitin E3 ligases. We reveal that the U-Box E3 ligase PUB17, a well-established positive regulator of multiple immune pathways in plants, is a target for degradation by the ubiquitin E3 ligase POB1. We also demonstrate that this targeted proteolysis occurs in the nuclei of plant cells. In this report we provide clear evidence that distinguishes between BTB domain dependant dimerization and Cullin-3 ubiquitin ligase assembly and link this to target degradation in plant immune signalling.

## Introduction

The capacity of plants to protect themselves against pathogens depends on detection mechanisms that recognize pathogen-derived molecules and then activate host defence responses. Plants have evolved an armoury of defence mechanisms that allow them to counter infection. These encompass both basal responses, triggered by recognition of conserved pathogen-associated molecular patterns (PAMPs), and pathogen-specific responses, mediated via pathogen- and plant-specific gene-for-gene recognition events. Recognition leads to activation of immune responses [1]. One feature of the immune response in plants is the hypersensitive response (HR). This involves a highly localized programmed cell death (PCD) of the infected region that helps to contain pathogen spread [2]. Control over HR-PCD is thus central to determining susceptibility or resistance to disease. Understanding how infected cells bring about downstream molecular signalling to establish and regulate cell death is a fundamental challenge in plant biology.

PCD also plays a critical role in development in plants and animals [3]. Therefore, understanding the mechanisms that control cell death has a significance that extends beyond plant-pathogen interactions.

It is well established in many organisms that ubiquitin (Ub) is a key modifier of signalling. The Ub-conjugation pathway involves the activity of 3 enzymes called E1 (Ub activase), E2 (Ub conjugase) and E3 (Ub ligase). The E3 facilitates the formation of an isopeptide linkage between Ub and the target protein. A polyUb chain is often formed by the addition of multiple Ub monomers. Polyubiquitination of a given substrate serves not only as a signal for degradation but also for targeting and re-profiling [4, 5]. Even the addition of a single ubiquitin moiety plays an important role in determining the fate of the substrate [6]. In this context, the critical role in substrate specificity resides mainly with the E3 ligase and these enzymes form the largest group of proteins within the Ub-enzyme cascade, indicating the presence of a large number of targets in the proteome. The *Arabidopsis* genome project identified more than a thousand genes encoding putative E3 ligases and these can be divided into seven classes which can be subdivided into two basic groups, dependant on the occurrence of either a HECT (Homology to E6-AP C-Terminus) or RING (Really Interesting New Gene)/ U-box domain [7]. RING containing proteins can either ubiquitinate substrates independently or function as part of a multi-subunit complex which, in plants, includes: SCF (Skp1-Cullin1-F-box), CUL3 (Cullin 3)-BTB/POZ (Bric a brac, Tramtrack and Broad complex/Pox virus and Zinc finger), CUL4-DDB1 (UV-Damaged DNA-Binding protein 1) and APC (Anaphase Promoting Complex).

Examples of E3 ligases from nearly all classes have been documented to be involved in immune responses against a range of pathogens in distantly related plant species, indicating plants have evolved to utilize the ubiquitin system for immunity against invading pathogens. A number of E3 ligases have been implicated in regulation of pathogen-associated molecular pattern (PAMP)-Triggered Immunity (PTI), activated in response to perception of conserved microbial molecules, and HR-PCD, which can be a component of PTI, but is often activated by perception of pathogen effector molecules (effector-triggered immunity, or ETI). Examples include the F-box protein COI1 that is required to initiate the defence response mediated by the phytohormone JA [8]. Similarly, another F-box protein, SON1 or RPD4, regulates defence responses independent of salicylic acid and systemic acquired resistance (SAR). Interestingly it is also targeted for destruction through ubiquitin-mediated and non-ubiquitin-mediated pathways [9]. F-box protein ACRE189 (or ACIF1) is required for Cf-4 and Cf-9 HR-PCD in tomato and tobacco. *Arabidopsis* homologs of ACIF1 act upon jasmonate and abscisic acid responsive gene pathways to promote defence [10].

Plant U-box (PUB) E3 ligases PUB12 and PUB13 work in concert to attenuate PTI by ubiquitinating the flg22 receptor FLS2, facilitating its degradation [11]. PUB13 regulates a number of processes, including flowering and senescence, in addition to immunity. Indeed, recent yeast-2-hybrid (Y2H) screens have revealed several potential substrates and binding partners for PUB13, including: phosphatidylinositol-4 kinase and RABA4B, with which it complexes to negatively regulate salicylic acid (SA)-mediated defences [12]; the transcription factor HFR1, involved in flowering [13]; and the ABA regulator ABI1, a protein phosphatase 2C family member, which is a PUB13 substrate for ubiquitination and degradation [14]. In addition to PUB12 and PUB13, PUBs 22, 23, and 24 also act in combination to negatively regulate plant immunity [15]. Single, double and triple *pub22 pub23 pub24* mutants exhibited progressively attenuated suppression of the flagellin-induced ROI burst, MPK3 activation and downstream PTI marker gene expression [15]. PUB22 attenuates PTI by targeting the exocyst component exo70B2 for ubiquitination and degradation. However there is very little information on the regulators or the substrates of U-box E3 ligases that positively regulate immunity.

PUB17 and its tobacco homolog ACRE276 (NtPUB17) were originally identified as positive regulators of defence against the fungal pathogen *Cladosporium fulvum* [16]. PUB17 functions in the plant nucleus to activate Cf-mediated HR-PCD [17]. A further member of this family, PUB20/CMPG1, is required for HR-PCD mediated by Avr9/Cf-9, Pto/AvrPto interactions, and recognition of the PAMP INF1 [18]. The RXLR effector AVR3a from *Phytophthora infestans* suppresses these HR-PCD events by stabilizing CMPG1 to prevent its normal activity [19, 20].

Here we report the identification and characterisation of a tobacco BTB domain E3 ligase protein, POB1, that functions to suppress HR-PCD during the plant immune response by targeting tobacco PUB17 for ubiquitin-mediated proteasomal degradation. Tobacco and *Nicotiana benthamiana* plants with reduced POB1 activity show accelerated HR-PCD mediated by a broad range of elicitors whilst those with increased POB1 levels suppress HR-PCD. We demonstrate that POB1 dimerization and nuclear localization are required for its function in HR-PCD suppression. Mutated versions of POB1 that show reduced interaction with PUB17 fail to suppress HR-PCD indicating that POB1-mediated degradation of a U-box E3 ligase is a critical step for regulating plant innate immunity.

## Results

### NtPUB17 physically interacts with a BTB-BACK domain containing protein, NtPOB1

**Fig 1:**
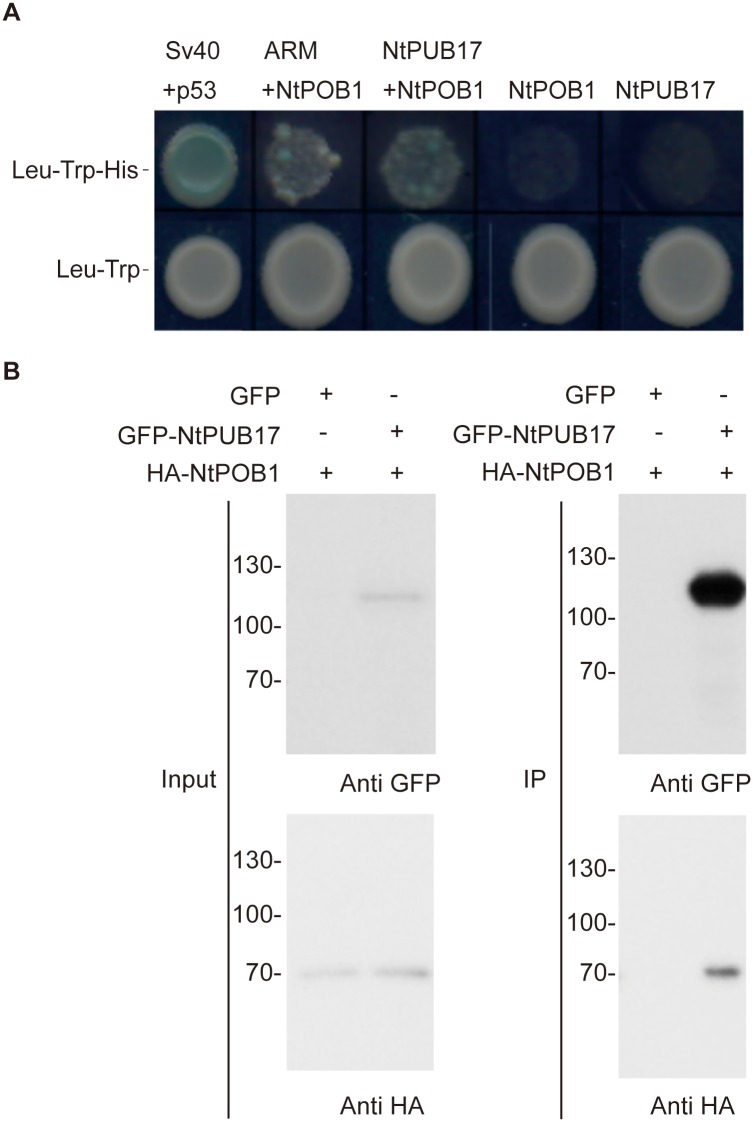
NtPUB17 directly interacts with NtPOB1. **A.** Yeast-2-Hybrid assays indicating that full length and ARM domain of NtACRE276 (NtPUB17) interact with NtPOB1. The various indicated proteins were expressed as AD- and BD- fusions in AH109 yeast cells. Transformed AH109 cells were grown on selective media (Leu^-^, Trp^-^, His^-^ and X-α-Gal) to test for interacting partners and on nonselective media (Leu^-^, Trp^-^) to test for the transformation efficiency. BD-SV40 and AD-p53 fusion proteins were used as positive controls for interaction in yeast. **B.** Immunoprecipitation analysis indicates that GFP-NtPUB17 and HA-NtPOB1 interact *in planta*. HA- and GFP fusions of NtPOB1 and PUB17 respectively were expressed in tobacco leaves using Agrobacterium-mediated transient assays. Total protein was extracted after 3 days and incubated with anti-GFP agarose beads to immunoprecipitate GFP-PUB17. The protein extracts (input) as well as the immunoprecipitates (IP) were analyzed by western blot using anti-HA and anti-GFP antibodies.

Recent yeast-2-hybrid (Y2H) screens with plant U-box proteins have identified a number of substrates for these E3 ubiquitin ligases [12, 13]. Therefore to identify potential substrates of the U-box protein NtPUB17 (Tobacco homolog of AtPUB17 and previously known as NtACRE276) we initiated a Y2H screen using the ARM repeat region of NtPUB17 (assumed to be the substrate-interacting domain) and identified a BTB domain protein which bore significant sequence homology to the Arabidopsis protein AtPOB1 (Fig 1A). We thus named this interactor NtPOB1. Co-immunoprecipitation studies using *Agrobacterium*-mediated transient assays (agroinfiltration) in *Nicotiana benthamiana* demonstrated that NtPUB17 interacts with NtPOB1 *in planta* (Fig 1B).

The BTB domain is an evolutionarily conserved, versatile protein-protein interaction motif that participates in a wide range of critical cellular functions in eukaryotes, including transcriptional regulation, cytoskeleton dynamics, ion channel assembly and gating, and targeting proteins for ubiquitination (reviewed in [21]). The *Arabidopsis* genome encodes 80 putative BTB domain genes, of which only a handful have been assigned function. These include the BTB ankyrin repeat domain protein NPR1 and the SA receptors NPR3 and NPR4 which regulate systemic acquired resistance (SAR) in plants [22].

Phylogenetic relationships between NtPOB1 and several representative BTB/POZ proteins are shown in S1A Fig Close analysis of the BTB/POZ protein superfamily indicated that BTB proteins could be classified into subfamilies based on additional motifs associated with the BTB/POZ domain. These motifs include the MATH, TRP, armadillo and ankyrin repeats, which are often involved in mediating protein-protein interactions. The predicted protein sequence of NtPOB1 identified an additional BTB-associated domain, called the BACK domain (S1B Fig). In other eukaryotes, proteins containing the BACK domain are always associated with a C-terminal KELCH domain [23]. However, interestingly, the BACK domain in NtPOB1 is not associated with any obvious KELCH-repeat and is therefore referred to as a BTB protein containing just an additional BACK domain.

Along with AtPOB1 and AtPOB2 there are another 9 BTB-BACK proteins in Arabidopsis, of which At4G01160 is the closest homolog to AtPOB1 and AtPOB2 (S1C Fig). BLAST searches using the *NtPOB1* cDNA sequence against the *N*. *benthamiana* genome sequence [24] identified several cDNA species with high similarity to *NtPOB1*. The *NtPOB1*-related *N*. *benthamiana* cDNA sequence fragments were used to isolate the full-length *NbPOB1* by RACE experiments [25]. The predicted protein sequence of NbPOB1 is 92% similar to NtPOB1. The phylogenetic tree in S1A Fig shows that NbPOB1 groups with AtPOB1, AtPOB2 and At4G01160, raising the possibility of functional conservation of these proteins between plants, similar to that demonstrated for AtPUB17 and NtPUB17, which were functionally exchangeable between tobacco and Arabidopsis [16].

### POB1 is a negative regulator of plant immunity

**Fig 2:**
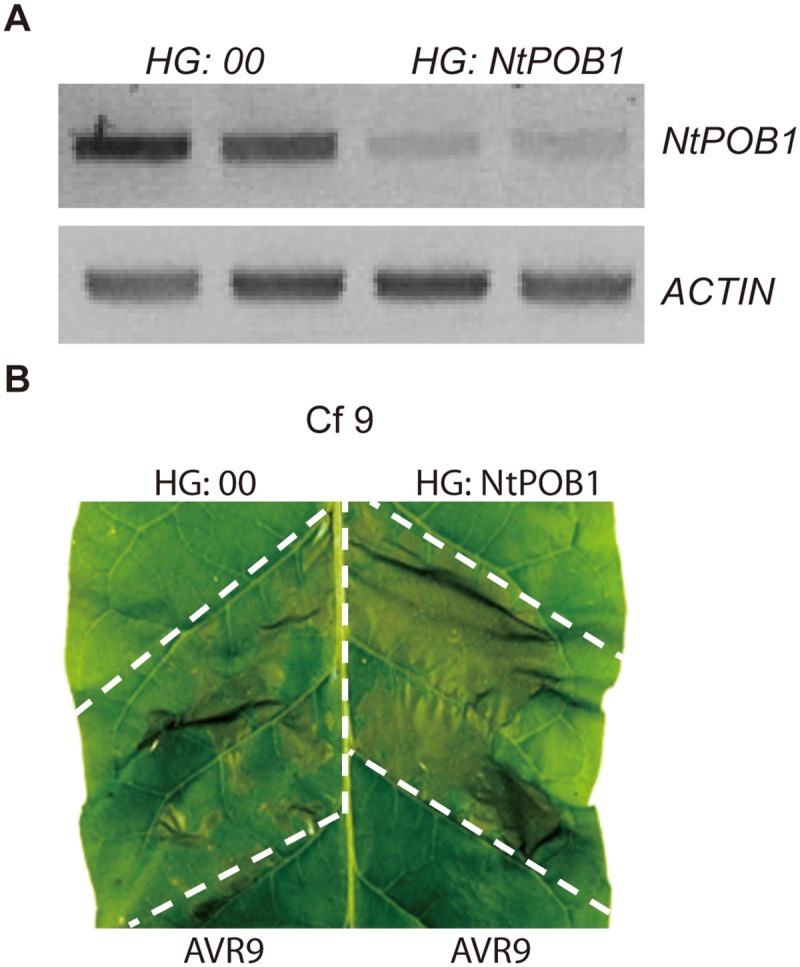
NtPOB1 is a negative regulator of avr9 –Cf9 mediated HR-PCD. **A.** RT-PCR analysis of the *NtPOB1* transcript levels in silenced and non-silenced tobacco plants 3 days after Agroinfiltrations with stated hairpin constructs. *ACTIN* transcript levels were used as loading controls. **B.** Leaf segments expressing vector only, HG: 00, or *NtPOB1*-silencing HG:NtPOB1 construct were infiltrated with Avr9 peptide 3 days after agroinfiltrations. Pictures were taken 36 hours after Avr9 treatment. At this time point *NtPOB1*-silenced leaf segments showed much earlier onset of HR-PCD than leaf segments expressing vector only. HR-PCD assays were repeated in at least three different leaves in three different *Cf-9* tobacco plants. Representative leaf image is shown of HR-PCD that was observed in 80% (8/10) of the leaves tested.

Previously we demonstrated that NtPUB17 acts as a positive regulator of Cf9-Avr9 mediated defence against *C*. *fulvum* [16]. Tobacco plants and cell cultures carrying a *Cf-9* transgene respond to Avr9 peptide with rapid induction of HR-PCD and associated responses in a strict gene-for-gene manner, indicating that all components required for efficient execution of the Avr9 effector triggered HR are present in this heterologous model system [26]. To assess whether tobacco *POB1 (NtPOB1)* plays a role in Avr9-mediated HR, hairpin (hp) RNA-mediated silencing was used to specifically knockdown *NtPOB1* gene expression in transgenic *Cf-9* tobacco plants [27]. Both HG: *NtPOB1* (*NtPOB1* specific hpRNA) and the control empty vector constructs were delivered into *Cf-9* tobacco leaves by agroinfiltration to transiently silence *NtPOB1* gene expression. *NtPOB1* transcript levels were analysed by RT-PCR three days following agroinfiltrations using *NtPOB1*-specific primers downstream of the expressed hpRNA fragment. A significant decrease in *NtPOB1* mRNA levels was observed in the leaves infiltrated with HG: NtPOB1 compared to the control leaves expressing the HG:00 empty vector construct (Fig 2A).

The HG: NtPOB1 and HG:00 constructs were expressed in the same leaf, opposite the main vein. Diluted Avr9 peptide stock solutions were infiltrated into the *Cf-9* tobacco leaf patches required to trigger limited HR in the control leaf segments. Silencing of *NtPOB1* triggered a more rapid *Cf-9*/*Avr9*-dependant HR while no visible HR was detectable in the control leaf segments at the same time point, suggesting that *NtPOB1* functions as a negative regulator of *Cf-9* mediated resistance (Fig 2B).

Virus-induced gene silencing (VIGS) [28, 29] was used to similarly study the function of *NbPOB1*. Gene silencing vectors based on the Tobacco Rattle Virus (TRV) [30–32] carrying 2 different and non-overlapping DNA fragments from the *NbPOB1* sequence were used to induce sequence-specific degradation of *NbPOB1*. BLAST analyses confirmed that both cDNA fragments (TRV: NbPOB1_A and TRV: NbPOB1_B) were unique to *NbPOB1*, ruling out potential silencing of closely related ‘off target’ genes. The efficiency and specificity of *NbPOB1* silencing was confirmed by RT-PCR on mRNA from emerging leaves of plants 21 days after inoculation with the TRV: NbPOB1_A and TRV: NbPOB1_B constructs (S2 and S3 Figs). *NbPOB1*-silenced plants had no obvious morphological differences to vector only control plants.

**Fig 3:**
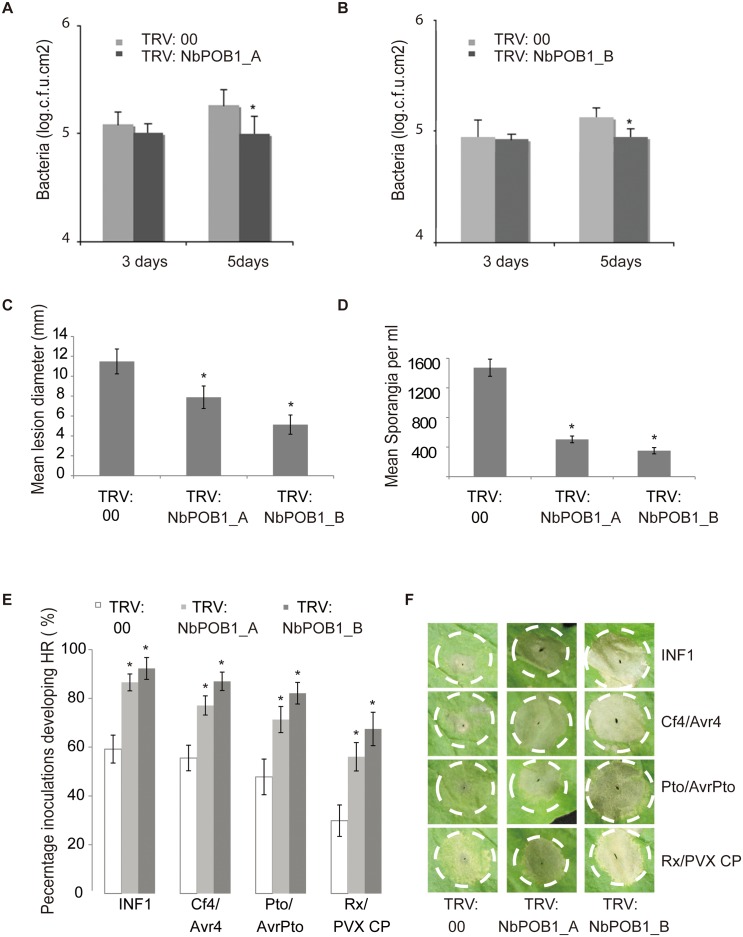
NbPOB1 is a negative regulator of basal defence and HR-PCD specified by diverse pathogen PAMPs and effectors. **A and B** Growth of *Ps pv*. *tabaci* in *NbPOB1*-silenced plants expressing TRV:00 (empty vector) or TRV:NbPOB1_A (**A**), or TRV:NbPOB1_B (**B**). Bacterial counts were performed 3 and 5 days post-inoculation (dpi). **C and D** *P infestans* infection assays indicating lesion growth measurement (**C**) and sporangia spore counts (**D**) in plants expressing TRV:00, TRV:NbPOB1_A or TRV:NbPOB1_B. **E.** Graphs show the percentage of infiltration sites developing clear HR-PCD (Y-axis) at 4 dpi of the PAMP INF1, and R genes/cognate effectors on *N*. *benthamiana* plants expressing TRV:00, TRV:NbPOB1_A and TRV:POB1_B. Experiments were repeated at least three times, each with 3 leaves from no less than 4 dedicated plants and error bars indicate +/- SE, * represents a P<0.05 statistically different (ANOVA) from TRV:GFP controls (n = 84 inoculations for INF1 on each of TRV:00, TRV:NbPOB1_A or TRV:NbPOB1_B; n = 84 for Pto/AvrPto; n = 78 for Cf4/Avr4; and n = 72 for Rx/PVX CP). **F** Photographs of typical infiltration zones at 4 dpi.

To ascertain the potential involvement of *NbPOB1* in immunity in *N*. *benthamiana*, pathogen infection studies were carried out with *Pseudomonas syringae* pv. *tabaci* (*Ps*. *tabaci*), a virulent pathogen of tobacco [33], on *NbPOB1*-silenced plants. A low titre of *Ps*. *tabaci* (1x10^4^ c.f.u.ml^-1^) was used to infect *N*. *benthamiana* plants that were inoculated with the *NbPOB1* silencing vectors TRV: NbPOB1_A and TRV: NbPOB1_B. Bacterial colony counts were performed 3 days and 5 days following *Ps*. *tabaci* infiltrations. No significant difference in bacterial growth was detected at 3 days post infection (dpi) between the control and the *NbPOB1*-silenced plants. However, the growth of the pathogen was significantly reduced in *NbPOB1-*silenced plants at 5 dpi, when compared to bacterial growth in the control plants (Fig 3A and 3B). In addition, *POB1*-silenced *N*. *benthamiana* plants were inoculated with the late blight pathogen *Phytophthora infestans*. *P*. *infestans* leaf colonisation was also significantly attenuated in *POB1* VIGS plants, measured as reduced lesion diameter and reduced development of sporangia (Fig 3C and 3D). These data indicate that plants down-regulated for *NbPOB1* transcript accumulation have enhanced capability of restricting both *Ps*. *tabaci* growth and *P*. *infestans* colonisation.

To further investigate the potential involvement of POB1 in negatively regulating immunity, a range of elicitors and gene-for-gene interactions that trigger HR-PCD were studied. Significantly, at an early stage of HR-PCD development in control plants (4 days post-inoculation), silencing *NbPOB1* by VIGS revealed accelerated HR-PCD induced by the *P*. *infestans* PAMP INF1, and by co-expression of tomato Cf4 and *C*. *fulvum* Avr4, tomato Pto and *P*. *syringae* AvrPto, and potato Rx with Potato Virus X coat protein (CP) (Fig 3E and 3F). These data indicate that POB1 negatively regulates multiple HR-PCD events.

### Over-expression of POB1 suppresses HR-PCD

Loss-of-function studies established that *NbPOB1* potentially negatively regulates HR-PCD triggered by the PAMP INF1 and by multiple gene-for-gene interactions. We examined the effect of over-expressing *NbPOB1* on disease resistance signalling by *Agrobacterium*-mediated transient over-expression in *Cf-9* tobacco.

**Fig 4:**
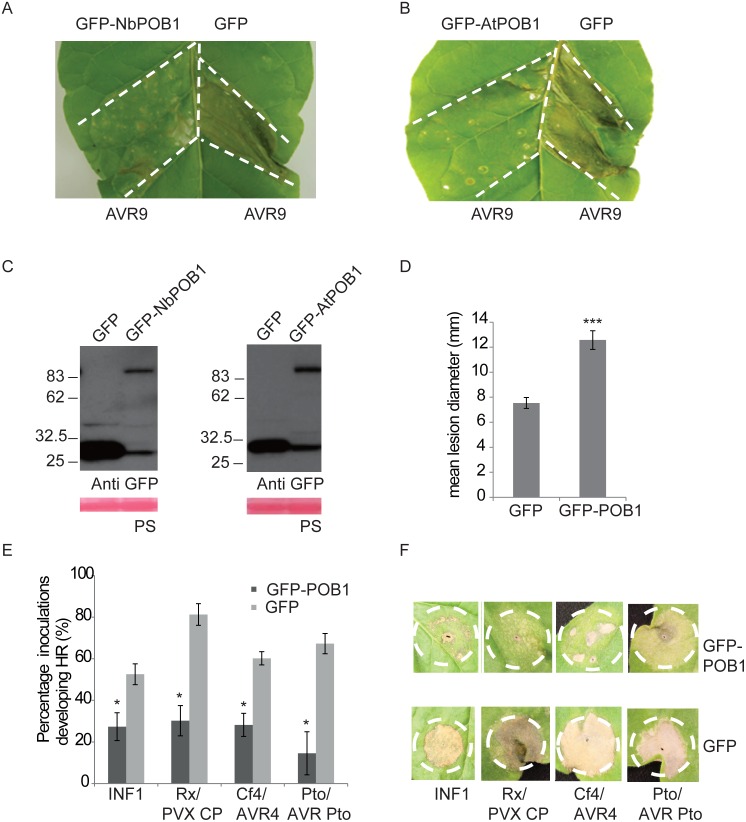
Over-expression of POB1 enhances *P*. *infestans* colonisation and suppresses HR-PCD. **A.** Over-expression of *N*. *benthamiana* NbPOB1 suppresses the generation of the Avr9-elicited HR cell death in Cf-9 tobacco plants. Agrobacteria containing the 35S: GFP-NbPOB1 and 35S:GFP were inoculated into opposing tobacco leaf segments (marked with white dotted line). Avr9 peptide was infiltrated into the leaf segments 3 days after Agroinfiltrations. HR-PCD assays were repeated at least in three different leaves each in three different *Cf-9* tobacco plants. Representative leaf image is shown that was observed in 70% (14/20) of the leaves tested. Pictures were taken 2 days after Avr9 treatment. **B** As in **A**, but expressing GFP-AtPOB1 fusion protein. Representative leaf image is shown that was observed in 70% (14/20) of the leaves tested. **C** Immunoblot analysis of NbPOB1and AtPOB1 in Cf-9 tobacco leaves. Total protein was extracted from leaves expressing GFP, GFP-NbPOB1 or GFP-AtPOB1, 3 days after Agroinfiltrations. Samples were immunoblotted with anti-GFP antibodies (top panel). Rubisco large subunit (rbcL) was used as a loading control as indicated by Ponceau staining (PS). **D**. Overexpression of GFP-POB1 in *N*.*benthamiana* enhances *P*. *infestans* leaf colonisation. Agroinfiltration was performed of either 35S: GFP-POB1 or 35S: GFP on opposing leaf segments of *N*. *benthamiana*. The leaves were then challenged with *P*. *infestans* 1 day after agro-infiltration. Quantification of lesion diameter 5dpi post inoculation. Statistical analysis was carried out using ANOVA with pairwise comparisons performed with a Holm-Sidak test; *** indicates p≤0.001 (n = 152); error bars show standard error. The graph represents the combined data from 4 biological replicates. **E** Graphs show the percentage of infiltration sites developing HR-PCD (Y-axis) at 4 days post-infiltration (dpi) of INF1 and *R* gene/cognate effector pairs, co-expressed with either 35S:GFP alone or 35S:GFP-POB1 on *N*. *benthamiana* plants. Experiments were repeated at least three times, each with no less than 8 dedicated plants (n = 132 inoculation sites for INF1; 108 for Rx/PVX; 124 for Cf4/Avr4; and 120 for Pto/AvrPto). Error bars indicate +/- SE and * represents a P<0.05 statistical difference (ANOVA) from GFP control. **F** Photographs of typical infiltration zones (white dotted lines) in **E** at 7 dpi.

*Cf-9* tobacco leaf sections were inoculated with *Agrobacterium* containing a construct expressing *NbPOB1* fused to the Green Fluorescent Protein (GFP) at its N-terminus. As a control, *Agrobacterium* containing GFP alone was used to inoculate the corresponding leaf sections on the other side of the main vein. Three days after Agroinfiltrations, the leaf segments were infiltrated with Avr9 peptide to induce *Cf-9*-dependant HR-PCD. Overexpression of *NbPOB1* suppressed the *Cf-9*/*Avr9*-mediated HR-PCD in 70% of leaves tested compared to the corresponding *GFP* control (Fig 4A).

Previously, we established that AtPUB17 was able to complement the pro-cell death function of NtPUB17 in tobacco lines where *NtPUB17* was silenced, establishing functional conservation between *Solanaceae* and *Brassicacea* species [16]. Given the high sequence similarity between AtPOB1 and NbPOB1 we tested whether AtPOB1 was also functionally interchangeable with NbPOB1 and was able to suppress *Cf-9* dependant HR. *Agrobacterium* containing the GFP-AtPOB1 construct was infiltrated into one side of *Cf-9* tobacco leaves. Three days after Agroinfiltrations the leaf segments were then infiltrated with Avr9 peptide to elicit HR. Leaf segments over-expressing the GFP-AtPOB1 fusion protein suppressed the formation of the HR cell death in 70% of the leaves tested, compared to the leaf segments expressing the GFP control (Fig 4B). Immunoblots indicated that both GFP-NbPOB1 and GFP-AtPOB1 were stable as intact fusion proteins *in planta* (Fig 4C). This indicates that AtPOB1 and NbPOB1 are functional orthologs (hereafter called POB1) acting as negative regulators of Avr9-elicited HR-PCD.

We tested whether over-expression of GFP-POB1 would enhance susceptibility to *P*. *infestans* on *N*. *benthamiana* leaves. Compared to the GFP control, GFP-POB1 expression resulted in significantly enhanced *P*. *infestans* leaf colonisation (Fig 4D).

Finally, we demonstrated the central role of POB1 by extending this HR-PCD suppression to different classes of cell death inducers in *N*. *benthamiana*. INF1, Cf4 with Avr4, Rx with PVX coat protein, and Pto with AvrPto, were each co-expressed with either GFP alone or GFP-POB1. In each case, HR-PCD was attenuated specifically when the HR-PCD elicitors were co-expressed with GFP-POB1 (Fig 4E and 4F). These data underline the importance of POB1 as a regulator of diverse defence pathways.

### Overexpression of POB1 destabilises PUB17 in the nucleoplasm

**Fig 5:**
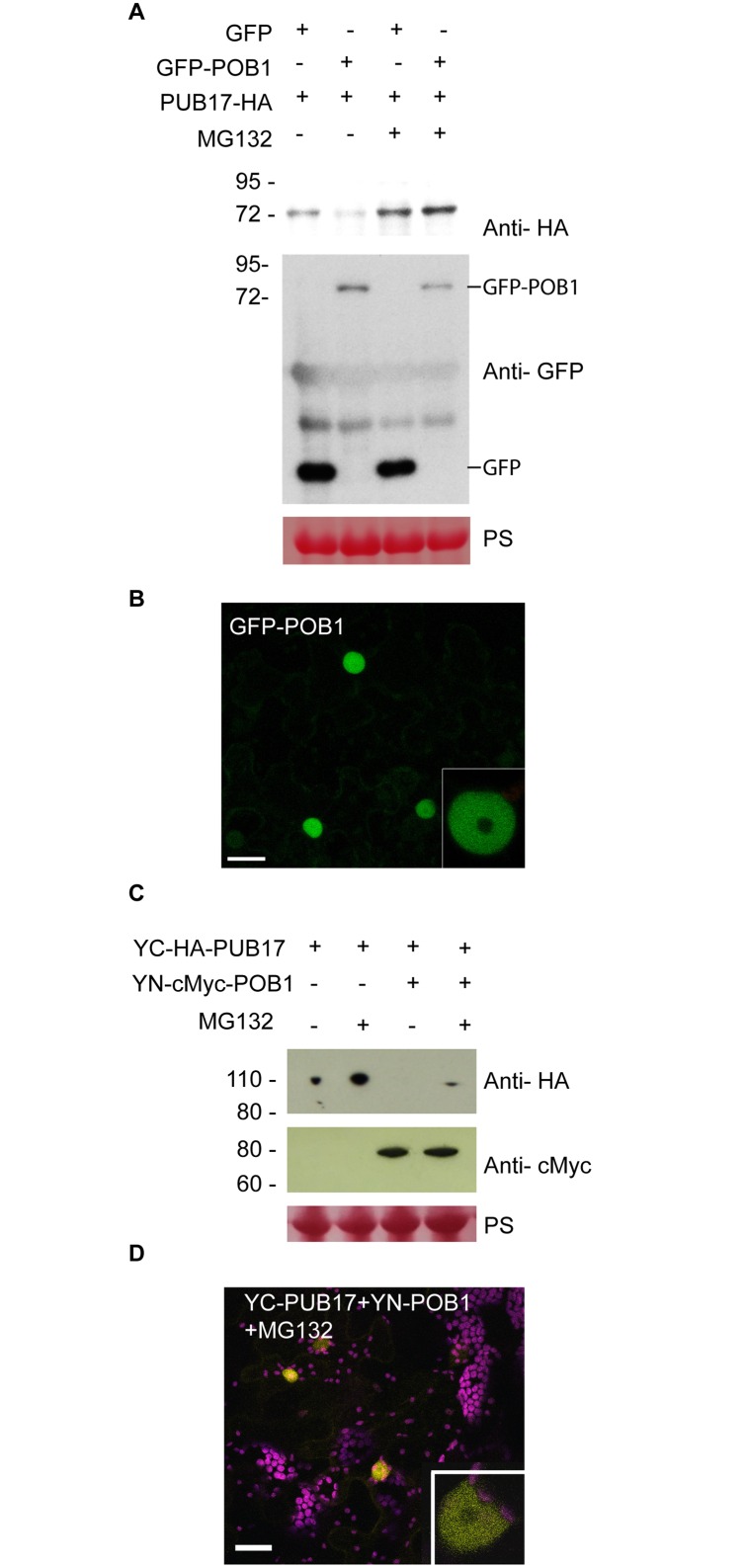
Overexpression of POB1 reduces PUB17 protein accumulation in nuclei of *N*. *benthamiana*. **A.** Western blot analysis indicates that GFP-POB1 overexpression destabilized HA-PUB17 *in planta*. HA- and GFP- fusions of PUB17 and POB1, respectively, along with GFP-only controls were expressed in *N*. *benthamiana* leaves using Agrobacterium-mediated transient assays. Total protein extracted after 3 days were analyzed by immunoblotting using anti-HA and anti-GFP antibodies. Size markers (kD) are shown to the left of each immunoblot. **B.** POB1 localizes in the nucleus. Images are confocal projections and the close up nuclear confocal images (inset) shows the nucleoplasmic localisation of GFP-POB1. *N*. *benthamiana* leaves were examined by confocal microscopy 48 h after infiltration of 35S-GFP-POB1 constructs. The scale bar is 20 μm. **C** PUB17 was destabilized by POB1. Transient expression of YC-PUB17 alone, or co-expressed with YN-POB1 in *N*. *benthamiana* leaves, with or without treatment with MG132. Immunoblots with an anti-HA antibody showing stable protein fusions of YC-PUB17 and the anti-myc antibody shows stable protein fusions of YN-POB1 of the expected size. The lower panels show Ponceau staining (PS) of the membrane as a loading control. **D.** POB1 interacts with PUB17 in the nucleus. Confocal image of bimolecular fluorescence complementation (BiFC) following co-expression of YN-POB1 and YC-PUB17 in the presence of MG132 reveals YFP fluorescence in the nucleoplasm. The scale bar is 20 μm.

To ascertain the biological significance of POB1-PUB17 interaction in defence suppression we investigated the levels of PUB17 and POB1 protein in *N*. *benthamiana* when they are co-expressed. We observed that the abundance of PUB17 protein was decreased when co-expressed with POB1 and this effect was mitigated when co-infiltrated with the proteasome inhibitor MG132 (Fig 5A). In contrast, silencing of *POB1* by VIGS resulted in enhanced accumulation of PUB17 protein (S4 Fig). Taken together with the yeast-two-hybrid and co-immunoprecipitation data (Fig 1) this indicated that PUB17 is a substrate for ubiquitination by POB1, leading to its proteasome-mediated degradation.

Transient expression assays in *N*. *benthamiana* indicated that GFP-POB1 was predominantly located in the nucleoplasm (Fig 5B), whilst PUB17 was shown previously to be located in the nucleoplasm and nucleolus [17]. We performed bimolecular fluorescence complementation (BiFC), fusing one half of YFP (YC) to the N-terminus of PUB17 and the other half (YN) to POB1. When expressed alone *in planta*, YC-PUB17 was detectable in immunoblots. However, when co-expressed with YN-POB1, YC-PUB17 was only detected in the presence of MG132 (Fig 5C). Confocal microscopy of *N*. *benthamiana* cells co-expressing YC-PUB17 and YN-POB1 in the presence of MG132 revealed reconstituted YFP fluorescence in the nucleoplasm, implicating this as the site for interaction between these proteins (Fig 5D).

**Fig 6:**
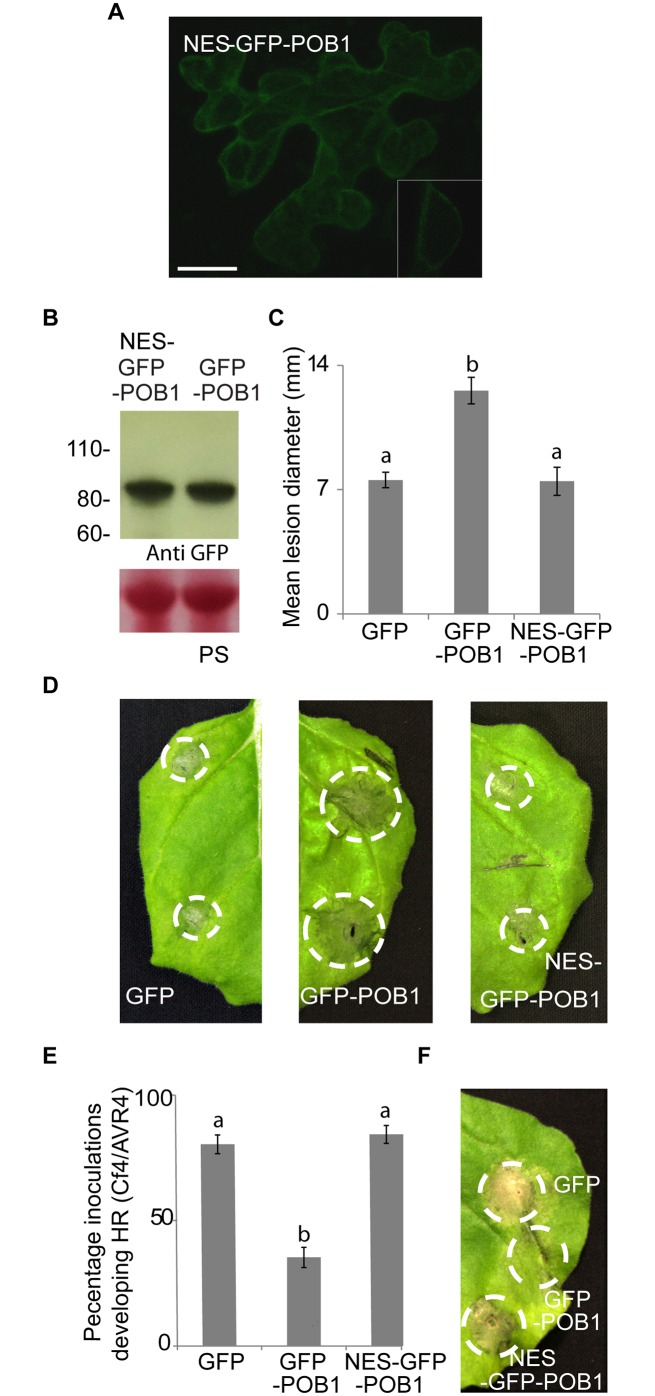
POB1 suppression of plant immunity occurs in plant nuclei. **A.** NES-GFP-POB1 was excluded from the nucleus. A representative *N*. *benthamiana* leaf expressing NES-GFP-POB1 examined by confocal microscopy 48 h after infiltration of NES-GFP-POB1, with close-up nuclear confocal image inset. The scale bar is 20 μm. **B.** GFP-POB1 and NES-GFP-POB1 fusion proteins are intact *in planta*. Immunoblots were probed with anti-GFP antibody. The lower panels show Ponceau staining of the membrane as a loading control (PS). **C.** GFP-POB1 significantly enhances *P*. *infestans* colonization (p≤0.001, n = 109), but NES-GFP-POB1 fails to do so. Mean lesion diameter of *P*. *infestans* colonisation following expression of GFP control, GFP-POB1 and NES-GFP-POB1.The lesion diameter was measured 5 days post inoculation. Statistical analysis was carried out using ANOVA with pairwise comparisons performed with a Holm-Sidak test. Error bars show standard error. The graph represents the combined data from 3 biological reps. **D.** Representative images of *P*. *infestans* colonisation in **C. E.** GFP-POB1 suppresses HR-PCD triggered by Cf4/Avr4, but NES-GFP-POB1 fails to suppress this (letters denote whether results are statistically significantly different, p≤0.001, n = 51). Mean % HR-PCD of Cf4/Avr4 co-expressed with GFP control, GFP-POB1 and NES-GFP-POB1 Statistical analysis was carried out using ANOVA with pairwise comparisons performed with a Holm-Sidak test; error bars show standard error. **F.** Representative images of HR-PCD in **E**.

To further investigate the importance of the nuclear localisation of POB1 for its function, a nuclear export signal (NES) was added to the N-terminus of GFP-POB1 to form NES-GFP-POB1. Transient expression of NES-GFP-POB1 in *N*. *benthamiana* leaves revealed that the fusion protein was stable and that GFP fluorescence was mainly located in the cytoplasm of the cells examined (Fig 6A and 6B). Crucially, in contrast to GFP-POB1, NES-GFP-POB1 expression failed to enhance *P*. *infestans* colonisation (Fig 6C and 6D) and failed also to suppress Cf4-Avr4-mediated cell death (Fig 6E and 6F). Our data indicates that the nuclear localisation of POB1 is required for its HR-PCD suppression function and this involves the degradation of PUB17.

### POB1 dimerization is critical for cell death suppression and PUB17 turnover

**Fig 7:**
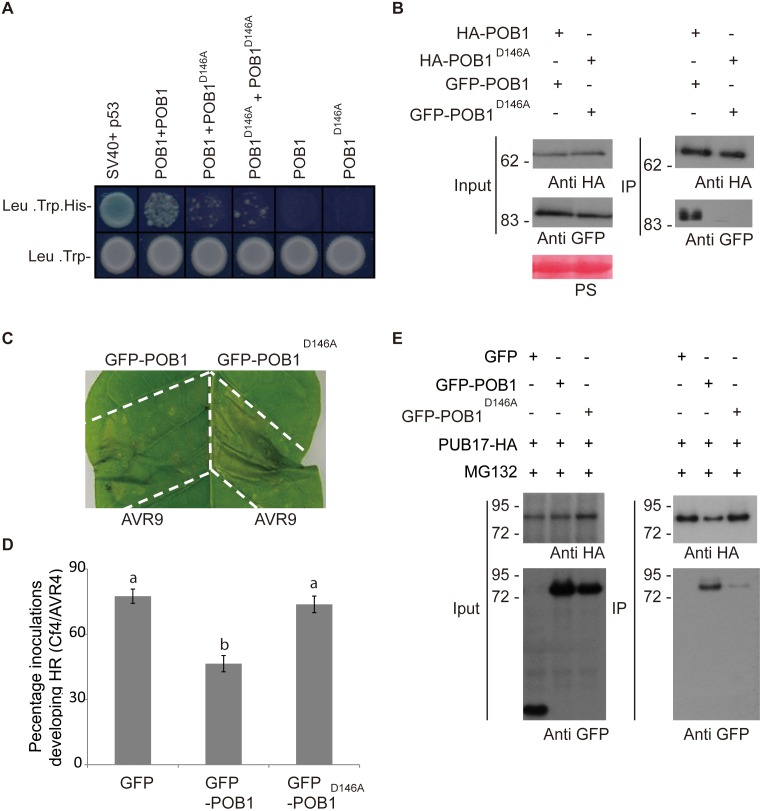
POB1 dimerisation is critical for cell death suppression and PUB17 turnover. **A.** POB1 D146A mutant protein cannot dimerize in yeast. Wildtype POB1 and POB1^D146A^ mutant proteins were expressed as AD- and BD- fusions in AH109 yeast cells. Transformed AH109 cells were grown on selective media (Leu-, Trp-, His- and X-α-Gal) to test for interacting partners and on nonselective media (Leu-, Trp-) to test for the transformation efficiency. BD-SV40 and AD-p53 fusion proteins were used as positive controls. **B** Immunoprecipitation analysis of the POB1^D146A^ mutation on POB1 dimerization. HA- and GFP fusions of the wild-type POB1 and mutant POB1 ^D146A^ were expressed in *N*. *benthamiana* leaves. The protein extracts and immunoprecipitates (IP) were analyzed by immunoblotting using anti-HA and anti-GFP antibodies. As indicated barely any HA-POB1^D146A^ co-immunoprecipitates with the GFP-POB1^D146A^. **C.** Transient over-expression of GFP-POB1 and GFP-POB1^D146A^ in Cf-9 tobacco. Agrobacteria containing the indicated constructs were infiltrated into Cf-9 tobacco leaf segments. Avr9 peptide was infiltrated into the leaf segments 3 days after agroinfiltrations. HR-PCD assays were repeated in at least three different leaves in three different *Cf-9* tobacco plants. Representative leaf image is shown where HR developed in leaves (9/20) expressing detectable AtPOB1 ^D146A^. Pictures were taken 2 days after Avr9 treatment. **D.** Graph of HR-PCD of Cf4/Avr4 co-expressed with GFP-POB1 or GFP-AtPOB1^D146A^. Statistical analysis was carried out using ANOVA with pairwise comparisons performed with a Holm-Sidak test; and letters denote whether results are statistically significantly different, p≤0.001 (n = 99); error bars show standard error. The graph represents the combined data from 4 biological reps. **E.** Immunoprecipitation analysis of the POB1^D146A^ mutation on POB1 interaction with PUB17. GFP fusions of the wild-type POB1 and mutant POB1 ^D146A^ were expressed in *N*. *benthamiana* leaves along with HA-PUB17 and treated with proteasome inhibitor MG132. The protein extracts (input) and immunoprecipitates (IP) were analyzed by immunoblotting using anti-HA and anti-GFP antibodies. As indicated barely any GFP-POB1^D146A^ immunoprecipitatied with PUB17-HA.

BTB proteins have been shown to form dimers in other eukaryotes [23]. However there is little *in vivo* evidence for the functional significance of dimerization of BTB domain proteins in plants. Alignment of several BTB/POZ domains from different proteins with the POB1 BTB/POZ domain identified a conserved Aspartate residue (D146). Mutation of the corresponding residue in PLZF (D35) exhibited reduced dimerization efficiency [34]. The D146 residue in POB1 was mutated to an alanine residue. The effect of this mutation on POB1 dimerization was tested using the yeast-two-hybrid system. These experiments demonstrate that the dimerization efficiency of POB1^D146A^ with itself or with POB1 is markedly reduced in yeast, compared to dimerization of POB1 with itself (Fig 7A).

To test whether this was the case also *in planta*, *Agrobacterium* containing HA-POB1 and GFP-POB1 and separately the D146A mutant versions (HA-POB1^D146A^ and GFP-POB1^D146A^) were co-expressed in tobacco leaves. Three days after Agroinfiltration, total protein was extracted and the expression of all protein was confirmed by immunoblotting with anti-HA and anti-GFP antibodies (Fig 7B). Both wild-type and mutant POB1 were expressed to the same level (Fig 7B), indicating that the D146A mutation did not affect the stability of POB1. Co-immunoprecipitations were performed using anti-HA antibodies to immunoprecipitate HA-POB1 and HA-POB1^D146A^. Whereas GFP-POB1 co-immunoprecipitated with HA-POB1, HA-POB1^D146A^ did not co-immunoprecipitate GFP-POB1^D146A^ (Fig 7B), confirming the effect of the D146A mutation in disrupting BTB/POZ-mediated dimerization *in planta*.

To examine the effect of the D146A mutation on the ability of POB1 to suppress *Cf-9/Avr9*-dependant HR-PCD, GFP-POB1^D146A^ and GFP-POB1 were transiently expressed in *Cf-9* tobacco leaves. Three days following Agroinfiltrations, Avr9 peptide was infiltrated into the leaf segments and suppression of HR-PCD was observed only in the segments expressing the wildtype GFP-POB1 and not in GFP-POB1^D146A^ segments (Fig 7C). Moreover, whereas overexpression of GFP-POB1 suppressed Cf4/Avr4-induced cell death, the GFP-POB1^D146A^ dimerization mutant was unable to suppress this cell death, when compared to the GFP control (Fig 7D), indicating that the dimerization of POB1 is essential for its function in suppressing HR-PCD.

Dimerization and formation of high order complexes are a characteristic of BTB/POZ proteins and are essential for their function in acting as E3 ligases [35]. As expected for BTB domain proteins, *N*. *benthamiana* transient assays followed by co-immunoprecipitation experiments revealed that HA-POB1 and HA-POB1^D146A^ were able to interact with GFP-tagged-CUL3 indicating that POB1 is able to form ubiquitin E3 ligase complexes *in planta* (S5 Fig). However the dimerization mutant GFP-POB1^D146A^ that is unable to suppress HR-PCD had significantly reduced capacity to co-immunoprecipitate PUB17. Moreover, PUB17 was more stable when co-expressed with GFP-POB1^D146A^ than with GFP-POB1 (Fig 7E). This indicates that suppression of cell death by POB1 is in part due to targeting PUB17 for proteasomal degradation.

## Discussion

Plant ubiquitin ligases regulate diverse aspects of plant development and morphogenesis, controlling multiple intracellular signalling pathways. The Plant U-box E3 ligase gene family has been shown to play critical roles in diverse processes. However, little information exists on how these E3 ligases are regulated at the protein level. Our study identified POB1, a BTB domain E3 ligase that targets PUB17 U-box protein for proteasomal degradation, thereby highlighting a novel mechanism for regulating the activity of U-box proteins.

Most BTB domain proteins can be sub-classified according to their additional C-terminal domain. However POB1 lacks any identifiable C-terminal domain but contains a highly conserved sequence of ~70 amino acids immediately following the BTB domain called a BACK domain. Structural information predicts that the BTB proteins might perform the combined functions of Cullin platform interaction and substrate binding roles of the SKP1 and the F-box subunits respectively of the Skp1-Cullin-F-box (SCF) E3 ligase [35]. In this context it is postulated that the BACK domain may serve as a bridge between Cullin interaction and substrate orientation for optimal ubiquitin attachment. In *Arabidopsis* there are 11 BTB-BACK domain proteins. The targets of any, to date, were unknown.

### POB1 is vital for HR-PCD events in addition to those regulated by PUB17

Previous biochemical characterization of the tobacco PUB17 provided clear evidence for its role in positively regulating HR-PCD mediated by Cf9-avr9 [16]. However, PUB17 does not play a role in regulating INF1-triggered HR-PCD [17]. Thus, the suppression of INF1-triggered HR-PCD by POB1 indicates that it negatively regulates immune responses in addition to those controlled by PUB17. PUB17 is thus unlikely to be the only positive regulator of immunity that is a target for POB1-mediated degradation, and further work is needed to identify additional candidate substrates for ubiquitination by POB1.

Strong evidence supporting our claim that POB1 is a conserved regulatory hub controlling HR-PCD comes from transient overexpression data. We show that, when overexpressed, AtPOB1 and NbPOB1 are functional homologs capable of efficiently suppressing HR-PCD mediated by PAMPs and effectors from evolutionarily diverse pathogens. These data suggest that POB1 is a rate-limiting factor for efficient signaling flux to activate HR-PCD.

Most negative regulators of cell death have proved to be indirect, as the lesions result from the perturbation of metabolic pathways [35, 36]. However *RIN4*, *BON1*, and *BAP1* are some examples of the negative regulators than directly activate specific gene function [16, 37–39]. We have demonstrated that *POB1* is a newly identified negative regulator that may be comparable to *RIN4*, *BON1 and BAP1*. However, more recently, a BTB protein called NRL1 has been shown to act as a negative regulator of immunity, suppressing INF1-triggered cell death. NRL1 acts as a so-called susceptibility (S) factor, as it is required for infection by the late blight pathogen *P*. *infestans* [40]. Unlike POB1, however, it does not negatively regulate such a wide range of immune pathways. In addition, whereas POB1 functions in the nucleus, NRL1 apparently acts at the plant plasma membrane [40]. Interestingly, an RXLR effector from *P*. *infestans* targets NRL1, presumably supporting or enhancing its role as a negative regulator of immunity [40]. It will be interesting to see whether POB1 is similarly targeted by pathogen effectors to support its role in suppressing immunity.

### Dimerization of POB1 is crucial for its function in HR-PCD

Dimerization and formation of high order complexes are a characteristic of BTB/POZ proteins and are essential for their function [34]. Deletion of the BTB domain of PLZF affected its transcriptional repression activity, causing cell cycle arrest [41]. The importance and conserved function of the BTB domain was also demonstrated by replacing the BTB domain of the human BCL-6 BTB domain fused to the *Drosophila* Tramtrack69 (ttk69) and therefore replacing its own BTB domain without disrupting its activity in neuronal photoreceptor cell differentiation [42].

To test the importance of POB1 dimerization in suppressing the Avr9-triggered HR, a point mutation was generated based on the 3D structural model of the PLZF BTB domain [41]. The conserved Aspartate residue in POB1 (D146; corresponding to D35 of PLZF) located in the charged pocket of the BTB fold was mutated to an Alanine residue, which therefore neutralized the negative charge of Aspartate. The mutation not only reduced the dimerization ability of POB1, but this mutant also failed to suppress the Avr9-triggered HR-PCD in *Cf-9* tobacco, indicating that the charged pocket of the BTB is important not only in mediating dimerization of POB1 but also for its function. It is remarkable that the point mutation in the BTB domain and the BTB deletion mutant were both unable to suppress HR-PCD, emphasizing the importance of an intact BTB domain for the activity of POB1.

The BTB fold is structurally similar to the linker proteins Skp1 and Elongin C of the SCF and ECS ubiquitin ligase complexes, respectively [43]. Skp1 links the scaffold CUL1 to substrate-specific adaptors (F-box), while ElonginC bridges CUL2 with SCOS-box proteins [44]. Several reports identified BTB-proteins as CUL3-interactors, where the BTB domain directly interacts with CUL3. In agreement with this we found that AtPOB1 interacts with AtCUL3A in yeast and *in planta*, indicating that it can form BTB-CUL3 ubiquitin ligases *in vivo* (S3A and S3B Fig). However the POB1 POB1^D146A^ mutant that was unable to suppress Avr-9 mediated HR-PCD was also found to interact with CUL3, indicating that dimerization was not required for CUL3 interaction. In mammals, the MATH-BTB protein SPOP has been linked to ubiquitination of MacroH2A, to regulate its deposition on the inactive X chromosome [45], and DAXX, to regulate transcriptional repression of proapoptotic proteins such as p53 [46]. It was shown that dimerization mutants of SPOP also co-purify with CUL3. However, the dimerization-defective SPOP is significantly impaired for ubiquitination activity on its substrate protein. To support a similar effect on POB1 we found that POB1^D146A^ mutant was less effective in binding to PUB17 and concomitantly less able to suppress HR-PCD indicating that PUB17 is indeed the substrate for POB1.

### POB1 role in HR-PCD occurs in the nucleus

We demonstrated nuclear localization of POB1 to be important for its function by fusing the N-terminal nuclear exclusion signal (NES) to the full-length POB1. The NES-AtPOB1 fusion protein was successfully excluded from the nucleus and it subsequently could not suppress HR-PCD elicited by Avr4 in *Cf-4 N*. *benthamiana* cells and fail to enhance *P*. *infestans* colonisation (Fig 6C and 6E). Previous work in plant innate immunity has indicated that subsets of BTB domain proteins are located within the nucleus. The best understood nuclear localized BTB protein in plant immunity is NPR1, the master coactivator of SAR in plants [47, 48]. Unlike POB1, NPR1 contains recognizable ankyrin repeats at its C-terminus and evidence suggests that, in the absence of salicylic acid (SA), NPR1 is continually degraded in the nucleus in a proteasome-dependent manner, probably via CUL3 binding [49]. However a portion of NPR1 is also located in the cytoplasm and during SAR this portion is recruited to the nucleus and interacts with CUL3 to possibly ubiquitinate and degrade transcriptional repressors to fully activate SA marker genes. Although we did not find any evidence for POB1 localization in the cytoplasm excluding POB1 prevented its interaction with PUB17 and also attenuated HR-PCD suppression indicating that the main function of POB1 occurs within the nucleus.

## Materials and Methods

### Plant materials and growth conditions

Tobacco plants were grown in environmentally controlled cabinets at 24°C with 16hr light and 8 hr dark cycles. The *Cf-9* transgenic *N*. *tabacum* cv *Petite Havana* line 34.1B were previously described [50]. *N*. *benthamiana* was grown in individual pots in a glasshouse at 22°C (16/8 h light/dark cycle) as described in Bos et al. [19].

### RT-PCR

Total RNA from two 2 cm diameter leaf discs was isolated using the Trizol Reagent (Sigma-Aldrich) method following the manufacturer’s instructions. First-strand cDNAs were synthesized from 2 ug of total RNA using Superscript II RNAse H_ Reverse Transcriptase (Invitrogen). cDNAs for tobacco and *Nicotiana benthamiana* POB1 were amplified by PCR using gene- specific primers (S1 Table). RACE experiments were performed using a GeneRacer RACE Ready cDNA Kit (Invitrogen, USA) and according to the manufacturer’s instructions but with gene-specific primers.

### Pathogenicity assays

The virulent strain *Pseudomonas syrinage* pv. *tabaci* EV (*Ps* pv. *tabaci*) [51] was used to infect *N*. *benthamiana* plants. The Bacterial strain was grown at 28°C on King’s B agar plates containing the appropriate antibiotics for 2 days. The bacteria were then grown overnight in liquid King’s B medium at 28°C with constant shaking (200 rpm), washed once, and resuspended in 10 mM MgCl_2_ to a final concentration adjusted to 1x10^5^ cfu/ml. The culture was then infiltrated into the leaves using a 1mL syringe. The trays were put under a humidifier and bacterial counts were performed after 3 and 5 days.

*Phytophthora infestans* strain 88069 was used for *N*. *benthamiana* infection and was cultured on rye agar at 19°C for 2 weeks. Plates were flooded with 3 mL of water and scraped with a glass rod to release sporangia. The resulting solution was collected in a falcon tube, Sporangia numbers were counted using a hemocytometer and adjusted to 60,000 per mL. The suspension was incubated in the refrigerator at 4°C for one to four hours to let the sporangial cytoplasm cleave and release zoospores. 10 mL droplets were inoculated onto the abaxial side of leaves of detached or intact *N*. *benthamiana* plants stored on moist tissue in sealed boxes. The boxes containing the infected plants were gently wrapped with plastic cling-film, covered with black plastic, and incubated overnight at 19 degrees Celsius after inoculation. The black plastic covers were removed the following morning, and disease incubation continued under 16/8 hours light/dark conditions. The disease phenotype was recorded by measuring the lesion diameter of the leaves from intact plants at 5 days post inoculation and 7 days for the detached leaves. Sporangia counts were performed on 7 dpi leaves from VIGSed plants which had been immersed in 2ml H_2_O and vortexed to release sporangia. A Haemocytometer was used to count the number of sporangia recovered from each leaf and was expressed as sporangia/ml.

### *Avr9*-mediated HR tests

Transgenic *Avr9 N*. *tabacum* plants express the *Cladosporium fulvum* Avr9 peptide in the intercellular fluids, which was extracted as described in [52]. Leaves of 7–8 week-old *Avr9* tobacco plants were cut into thin strips, immersed into distilled water and subjected to a vacuum. Following release of the vacuum, the leaf strips were put in 20mL syringes attached to 50mL falcon tubes and centrifuged to discharge the intercellular fluid containing the Avr9 peptide. Several dilutions of the Avr9 peptide were used to trigger cell death in 7-week-old *Cf-9* tobacco [53]. Confocal microscopy

### Confocal microscopy

The target proteins were expressed in *N*. *benthamiana* leaves by Agrobacterium-mediated transient expression. *A*. *tumefacien* containing target protein fusions were pressure infiltrated into leaves of 4-week old *N*. *benthamiana* plants. *N*. *benthamiana* leaf cells expressing fluorescent protein fusions were imaged no later than 2 days after agroinfiltration by using a Leica TCS-SP2 AOBS confocal microscope. GFP was excited with 488 nm from an argon laser and its emissions were detected between 500 and 530 nm. Split-YFP was excited with 514 nm from an argon laser with emissions collected between 530–575 nm. Images were only collected from leaf cells expressing low levels of the protein fusions to minimise possible artefacts of ectopic protein expression. MG132 (100 μM) treatments were infiltrated 5 hours before imaging under the confocal microscope. Transient assays in *N*. *benthamiana* and *N*. *tabacum*.

### Transient assays

Tobacco transient assays were performed as described in [53]. *Agrobacteria* containing the desired construct were grown on LB plates at 28°C for 2 days. A few colonies were then picked and grown overnight at 28°C with constant shaking (200 rpm) in 10mL LB culture vials containing the appropriate antibiotics. The bacteria was pelleted and washed once with 10mM MgCl_2_. The cells were then resuspended in 10mM MgCl_2_ containing 200μM acetosyringone and the OD_600_ was adjusted to 0.2. The cultures were left for 2–3 hours at room temperature before infiltration. The *Agrobacteria* cultures were infiltrated into *N*. *benthamina* leaves by pressure-infiltration on the lower side of the leaves using a 1mL syringe. As for *Nicotiana tabacum*, infiltrations were carried out through small incisions on the lower side of the leaves (using a clean razor blade). The infiltrated plants were then put in a controlled growth cabinet at 22°C under constant light for 2–3 days to allow gene expression.

### Virus-induced gene silencing

Loss-of-function studies in *N*. *benthamiana* were carried out through the virus-induced gene silencing (VIGS) system [54] based on the Tobacco Rattle Virus (TRV) strain PPK20 [55]. The TRV genome consists of 2 RNAs. RNA1 (involved in movement) and RNA2 (involved in transmissibility). RNA1 and RNA2 are encoded by the *pBINTRA6* and *pTV00* respectively. pBINTRA6 was transformed into the *Agrobacterium* strain *LB440*, whereas *pTV00* (including the target sequence) was transformed into the *Agrobacterium* strain GV3101 containing the helper plasmid *pSA-rep*. *Agrobacteria* containing *pBINTRA6* and *pTV00* constructs were grown overnight in 10mL YEB cultures at 28°C with constant shaking (200 rpm). The cultures were then spun at 4000rpm for 10 minutes, washed once, and resuspended in 10mM MgCl_2_ containing 200μM acetosyringone to a final OD_600_ of 0.5. The *Agrobacteria* containing the respective pTV00 constructs were mixed with the *Agrobacteria* containing the *pBINTRA6* vector in a 1:1 ratio and incubated at room temperature in the dark for 2 hours. *NbPOB1* gene was amplified by PCR using gene- specific primers (S1 Table). A 426bp POB1_A fragment and a 732bp POB1_B fragment were amplified using primers described in S1 Table from *N*. *benthamiana* and cloned into pTV00 VIGS vector. The sequences used to silence NbPOB1_A and NbPOB1_B do not share homology with any other sequence annotated in the *N*. *benthamiana* genome. A pTV00 construct expressing GFP, was used as a control The mixed cultures were then used to inoculate 3–4 weeks old *N*. *benthamiana* plants by pressure infiltration at the lower side of the leaves. Inoculated plants were put in the growth rooms and grown for ~3 weeks until the onset of gene silencing. The silencing level of NbPOB1 was analysed by RT-PCR two to three weeks post infiltration. The silenced leaves at 3 weeks post-infiltration were used for pathogenicity assays.

### Transient gene-silencing in *Nicotiana tabacum*

The gene-silencing vector *pHELLSGATE12* [27] induces gene silencing transiently in *N*. *tabacum*, 3–4 days after *Agrobacterium* infiltrations through hairpin RNA (hpRNA)-mediated silencing. The *pHELLSGATE12* vector uses the Gateway technology (Invitrogen) to facilitate cloning of the insert in sense and anti-sense directions, separated by the *PDK* and *Catalase-1* introns. An entry clone contain 732bp of *NtPOB1* gene sequence (amplified by gene specific primers indicated in S1 Table) were mixed with the pHELLSGATE12 vector in the presence of LR clonase enzyme mix (as described in manufacturers instructions) and incubated at 25°C for 2h to overnight. The pHELLSGATE12 vector with the target sequence was next transformed into the Agrobacterium strain GV3101 by electroporation. Transformed *Agrobacteria* were grown overnight in 10mL LB cultures with the appropriate antibiotics (Rifampicin 100μg/ml and Spectinomycin 50μg/ml) at 28°C with constant shaking (220rpm). The cultures were next spun (2500 rpm, 10minutes), washed once and resuspended in 10mM MgCl_2_. The OD_600_ was adjusted to 0.5 and the cultures were incubated at room temperature before infiltration into *Cf-9 N*. *tabacum* leaves, allowing 3–4 days for *hpRNA* constructs to be generated, and consequently silencing of the target gene. The silencing level of NtPOB1 was analysed by RT-PCR using gene specific primers (S1 Table) 4 days post Agroinfiltration. The sequence used to silence *NtPOB1* recognizes both NtPOB1 isoforms in tobacco since they share 99% homology.

### Site-directed mutagenesis of plasmid DNA

POB1^D146A^ Site-directed mutagenesis was carried out using the QuickChange Site-Directed Mutagenesis Kit (Stratagene, U.S.A.) by using PfuTurbo DNA polymerase and pTOP-POB1 as a template. Primers containing the desired mutation were designed accordingly that are shown in S1 Table. Parental (i. e., the nonmutated) supercoiled dsDNA was removed by DpnI treatment. The resultant Ile-146 was verified by sequencing. One conserved amino acids of POB1, Aspartate at positions 146, were substituted with alanine, resulting in the mutant POB1^D146A^. The mutant POB1^D146A^ was recombined, using LR clonase, into pB7WGF2 or HA for in planta assays.

### Plant total protein extraction

Plant tissue from *Arabidopsis* and tobacco was ground in ice-cold homogenization buffer [25mM Tris-HCl, 150mM NaCl, 1mM EDTA, 10% (v/v) glycerol, 5mM DTT, 0.1% Triton X-100, Complete EDTA-free protease inhibitor tablet cocktail] using a mortar and pestle and clarified by centrifugation (12000g, 4°C, 15minutes). The supernatant was transferred to a clean Eppendorf tube and protein concentrations were determined using the Bradford assay (BioRad). The total protein fraction was then mixed with 1x SDS loading buffer [25mM Tris-HCl pH6.8, 10% (v/v) glycerol, 2% (w/v) SDS, 5% (v/v) β-mercaptoethanol, 0.001% (w/v) Bromophenol blue] and boiled for 5 minutes before being separated by SDS-PAGE.

### Protein quantification using the Braford assay

2μl of protein extract were mixed with 18μl sterile water and 900μl diluted (5-fold) Bradford reagent (BioRad). The mixture was then transferred to a cuvette and incubated at room temperature for 5min. The absorbance of the samples was read at a wavelength of 595, against a blank sample (no protein extract added). The concentration of the samples was calculated based on a standard curve.

### Immunoprecipitation of tagged proteins from *Nicotiana benthamiana* plant extract

Protein interactions were tested by expressing the proteins of interest transiently in *N*. *benthamiana* through Agroinfiltration. Three days after infiltration, 3–4 leaves were ground in 1ml of ice-cold extraction buffer using a pestle and mortar. The homogenate was spun at 12,000 rpm at 4°C and the supernatant was applied to a Sephadex-G25 (Sigma) column that had been pre-equilibrated in lysis buffer. Fifty μl of epitope antibody agarose conjugates (also pre-equilibrated in lysis buffer) were added to the protein samples and were left to rotate for 3–4 hours at 4°C. The agarose beads were then pelleted by centrifugation at 1,000g for 10sec at 4°C and washed 3 times with 1mL lysis buffer. After the final wash, 50μl of 1x SDS-loading buffer was added to the beads, boiled for 5min and the supernatant analyzed by SDS-PAGE.

### Western blotting and immunolabelling

After the SDS-PAGE run, proteins were transferred to a PVDF membrane (BioRad) using the Mini-PROTEAN Trans-Blot transfer cassette (BioRad) filled with cold transfer buffer [25mM Tris-HCl, 190mM glycine, 20% (v/v) methanol]. Electro-transfer of proteins to nitrocellulose membranes was carried out at 100V for 1h or at 30V overnight. The membrane was then blocked in 5% (w/v) non-fat dried milk dissolved in TBS-T [15mM Tris-HCl pH7.6, 150mM NaCl, 0.1% (v/v) Tween 20] with gentle agitation to reduce non-specific binding. Then primary antibodies diluted in TBS-T containing 5% (w/v) non-fat dried milk were added to membrane and incubated for 1–3 hours. Membranes were then washed several times with TBS-T for a total of 20min before incubation for 45 min with secondary HRP-conjugated antibodies. Following incubation, the membranes were washed five times with TBS-T for a total of 25min. Immunodetection was carried out using Immobilon (Millipore) for chemiluminescent detection in accordance with the manufacturer’s instructions. The membrane, washed from any unbound HRP-conjugated secondary antibody, was incubated in 1:1 Luminol Reagent and Peroxide Solution for 5 minutes at room temperature. Excess substrate was drained and the membrane was covered in cling film and placed in an X-ray cassette. The membrane was then exposed to X-ray film (Kodak) under safe red light conditions. The film was developed using the X-OMAT developing system (FUJI).

### Yeast-two-hybrid

Small-scale transformation of yeast was performed as described in the HybriZAP-2.1 Two-Hybrid Libraries instruction manual (Stratagene). A few colonies of AH109 cells growing on YPD agar (BD Biosciences) plates were inoculated in 10mL YPD medium (BD Biosciences) and grown overnight at 30°C with constant shaking (200rpm). The culture were then added to a fresh 100mL YPD medium and incubated for an additional 4–5 hours until the OD_600_ reached 0.8. At this stage, the yeast cells were spun at 1,000g for 5min, washed once in sterile water, and resuspended in 1mL LiAc/TE (0.1M LiAc and 10mM TE pH8.0) solution. Approximately 200ng of bait and prey plasmids were mixed with 100μg of pre-heated salmon testes DNA in an eppendorf tube. Hundred μl of the yeast cell suspension and 600μl of transformation solution [40% (w/v) PEG, 0.1M LiAc, 10mM TE pH8.0] were next added to the mixture and mixed by vortexing, followed by an incubation period of 30 min at 30°C with constant shaking (200 rpm). Seventy μl of DMSO was added to the transformation mix, mixed gently by inverting, heat-shocked at 42°C for 15min and then placed on ice for 10min. The cells were pelleted by centrifugation at maximum speed for 10sec and resuspended in 200μl sterile water. The cells were plated on Minimal SD agar Base medium (BD Biosciences) containing Leu^-^/Trp^-^ DO Supplement (BD Biosciences) as a selective medium for plasmid DNA co-transformation or Leu^-^/Trp^-^/His^-^ SO Supplement (BD Biosciences) with 20mM X-α-Gal (Melford) as a selective medium for testing interacting partners. The plates were incubated at 30°C for 3 to 5 days until colonies developed.

### Sequence analysis

Full-length protein sequences of Arabidopsis POB and BTB-containing genes, Mouse KEAP1 and NbPOB1 were aligned using MAFFT [56]. Up to 1000 iterations were allowed, and post-alignment local pairwise refinement was employed. Amino acid sequences of BTB and BACK domains (where present) from the above genes were also aligned separately using the same method, and manually refined in the case of the BACK domain alignment. Arabidopsis gene At2G30600 contains two BTB domains so both were included separately in the BTB alignment. Phylogenies were estimated separately from the BTB and BACK multiple alignments using the maximum likelihood (ML) method in MEGA5 [57]. Phylogenies were bootstrapped using 1000 replicates, Jones-Taylor-Thornton evolutionary model was used, and 5 gamma-distributed variation rates among sites were allowed. Gaps were treated by deletion. Nearest-neighbour interchange and automatic initial tree inference were used. Phylogenies were rooted using the well-established relevant domain from Mouse KEAP1 as outgroup. For comparison, phylogenies were also estimated from both sets of domain alignments in MEGA5 using maximum parsimony (MP), minimum evolution (ME), neighbor joining (NJ), and UPGMA methods with default parameters.

The domains on proteins were identified based on models from Prosite, Panther, Pfam, Smart, Superfam and Gene3D [58; 59, 60] and manually refined.

### Accession numbers

Sequence data for the *Nicotiana Benthamiana POB1* has been deposited in the GenBank data library under the accession number 1399145.

## Supporting Information

S1 Fig Sequence analysis reveals that AtPOB1 is a BTB-BACK protein. All proteins are from *Arabidopsis thaliana* (At) with the exception of NbPOB1 which is from *Nicotiana benthemiana* (Nb), NtPOB1 which is from *Nicotiana tobacco* (Nt), and MmKeap1 which is from *Mus musculus* (Mm). A. Dendrogram illustrating the grouping of AtPOB1, AtPOB2, NtPOB1 and NbPOB1 proteins and divergence from other BTB and BACK domain proteins. The dendrogram was inferred using the UPGMA method [61]. The optimal tree with the sum of branch length = 2.80878055 is shown. The tree is drawn to scale, with branch lengths in the same units as those of the evolutionary distances used to infer the phylogenetic tree. The evolutionary distances were computed using the Poisson correction method and are in the units of the number of amino acid substitutions per site. The analysis involved 12 amino acid sequences. All positions containing gaps and missing data were eliminated. There were a total of 236 positions in the final dataset. Evolutionary analyses were conducted in MEGA7. B. and C. Multiple sequence alignment of the domains from AtPOB1, AtPOB2 and NbPOB1 with B. BTB domains C. BACK domains from other *Arabidopsis* proteins and MmKeap1. The alignments were done in ClustalX 2.0.12. Alignment annotations: “*” represents full conservation, “:” represents strong conservation within a ‘strong’ group and “.” represents conservation within a ‘weak’ group.
(TIF)

S2 Fig Quantitative Real-Time PCR analysis of NbPOB1 levels in VIGS plants. RT-PCR analysis of *NbPOB1* transcript levels in experiments for *Ps*. *tabaci* infecting assays. Transcripts were analyzed 21 days after Agroinoculations with *TRV*:*00* and *TRV*:*NbPOB1_A* (A) and *TRV*:*NbPOB1_B* (B). ACTIN transcript levels were used as loading controls.
(TIF)

S3 Fig Semi-quantitative RT-PCR analysis of transcripts of the most closely related POB1-like gene in NbPOB1 VIGS plants indicates that silencing is specific to POB1 gene expression. RT-PCR analysis of mRNA from of VIGs plants expressing TRV:00 (empty vector) or TRV:NbPOB1_B (POB1-specific). Transcripts were analysed 21 days after gene silencing. To confirm TRV:NbPOB1_B specificity, the expression level of the most related gene (*POB1-like*; EST: Niben101Scf00503g10005.1) was analysed by RT-PCR. *NbACTIN* transcript levels were used as loading controls.
(TIF)

S4 Fig Silencing of *NbPOB1* leads to the accumulation of PUB17 protein in *N*. *Benthamiana*. A. Western blot analysis indicates that NbPOB1-silenced plants accumulate HA-PUB17 in plants. HA-tagged PUB17 driven by the CAMV 35S promoter was transiently expressed in *NbPOB1-*silenced (TRV:NbPOB1_B) plants or empty vector control (TRV:00). Total protein was extracted after 3 days and HA-PUB17 protein levels were examined by immunoblotting with anti-HA antibodies. Rubisco was used as a loading control as indicated by Ponceau staining (PS). B. RT-PCR analysis indicates that *HA-PUB17* gene expression is equal in both empty vector and *NbPOB1* silenced plants.
(TIF)

S5 Fig AtPOB1 dimer mutants that cannot suppress HR-PCD maintain interaction with AtCUL3A. A. AtPOB1 interacts with AtCUL3A in yeast. AtPOB1 and AtCUL3A were expressed as binding domain (BD) and activation (AD)-fusion proteins respectively. Transformed AH109 cells were grown on triple selective media (Leu-, Trp-, His-) containing X-α-Gal to test for interacting partners and on double selective media (Leu-, Trp-) to test for the transformation efficiency. B. AtPOB1 interacts with AtCUL3A in planta. Agrobacteria containing 35S:GFP-AtCUL3A and 35S:HA-AtPOB1 or 35S:HA-AtPOB1^D146A^ were co-infiltrated into tobacco leaves. As a control, the 35S:HA-AtPOB1 construct was co-infiltrated with 35S:GFP. The total protein extracts and the immunoprecipitate fractions were analyzed by western blot and probed with anti-GFP and anti-HA antibodies. Bottom panel, rubisco large subunit is loading control (PS).
(TIF)

S1 Table DNA Oligonucleotide primers used in the study. (XLSX)

S2 Table DNA constructs generated as part of this study. (DOCX)
