# Supplementary figures and images for: BTB-BACK Domain Protein POB1 Suppresses Immune Cell Death by Targeting Ubiquitin E3 ligase PUB17 for Degradation
Source: PLoS Genet. 2017 Jan 5;13(1):e1006540. doi: 10.1371/journal.pgen.1006540 (PMC5249250; doi:10.1371/journal.pgen.1006540)

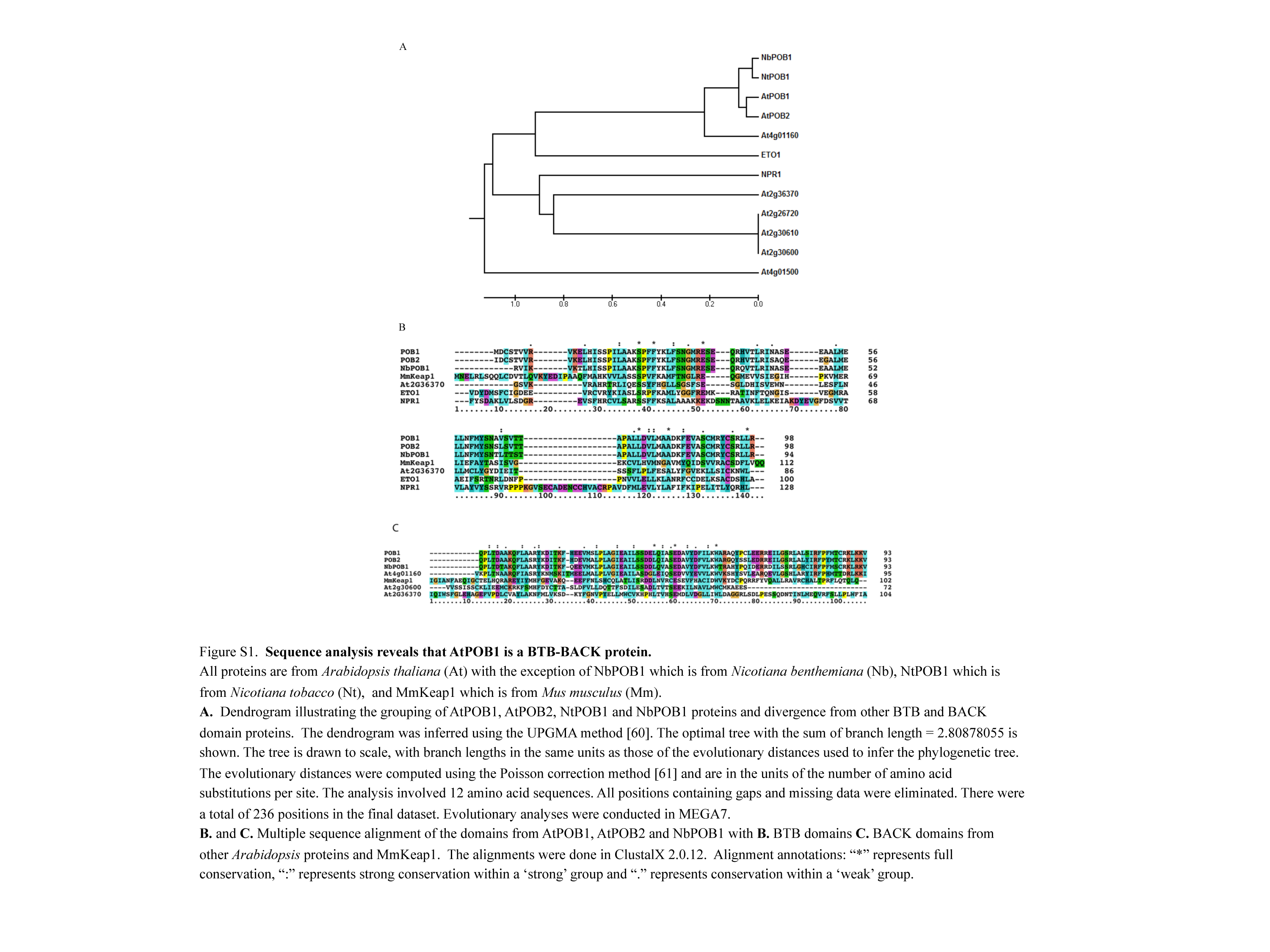

Supplement: S1 Fig — All proteins are from Arabidopsis thaliana (At) with the exception of NbPOB1 which is from Nicotiana benthemiana (Nb), NtPOB1 which is from Nicotiana tobacco (Nt), and MmKeap1 which is from Mus musculus (Mm). A. Dendrogram illustrating the grouping of AtPOB1, AtPOB2, NtPOB1 and NbPOB1 proteins and divergence from other BTB and BACK domain proteins. The dendrogram was inferred using the UPGMA method [61]. The optimal tree with the sum of branch length = 2.80878055 is shown. The tree is drawn to scale, with branch lengths in the same units as those of the evolutionary distances used to infer the phylogenetic tree. The evolutionary distances were computed using the Poisson correction method and are in the units of the number of amino acid substitutions per site. The analysis involved 12 amino acid sequences. All positions containing gaps and missing data were eliminated. There were a total of 236 positions in the final dataset. Evolutionary analyses were conducted in MEGA7. B. and C. Multiple sequence alignment of the domains from AtPOB1, AtPOB2 and NbPOB1 with B. BTB domains C. BACK domains from other Arabidopsis proteins and MmKeap1. The alignments were done in ClustalX 2.0.12. Alignment annotations: “*” represents full conservation, “:” represents strong conservation within a ‘strong’ group and “.” represents conservation within a ‘weak’ group. (TIF) [file pgen.1006540.s001.tif]

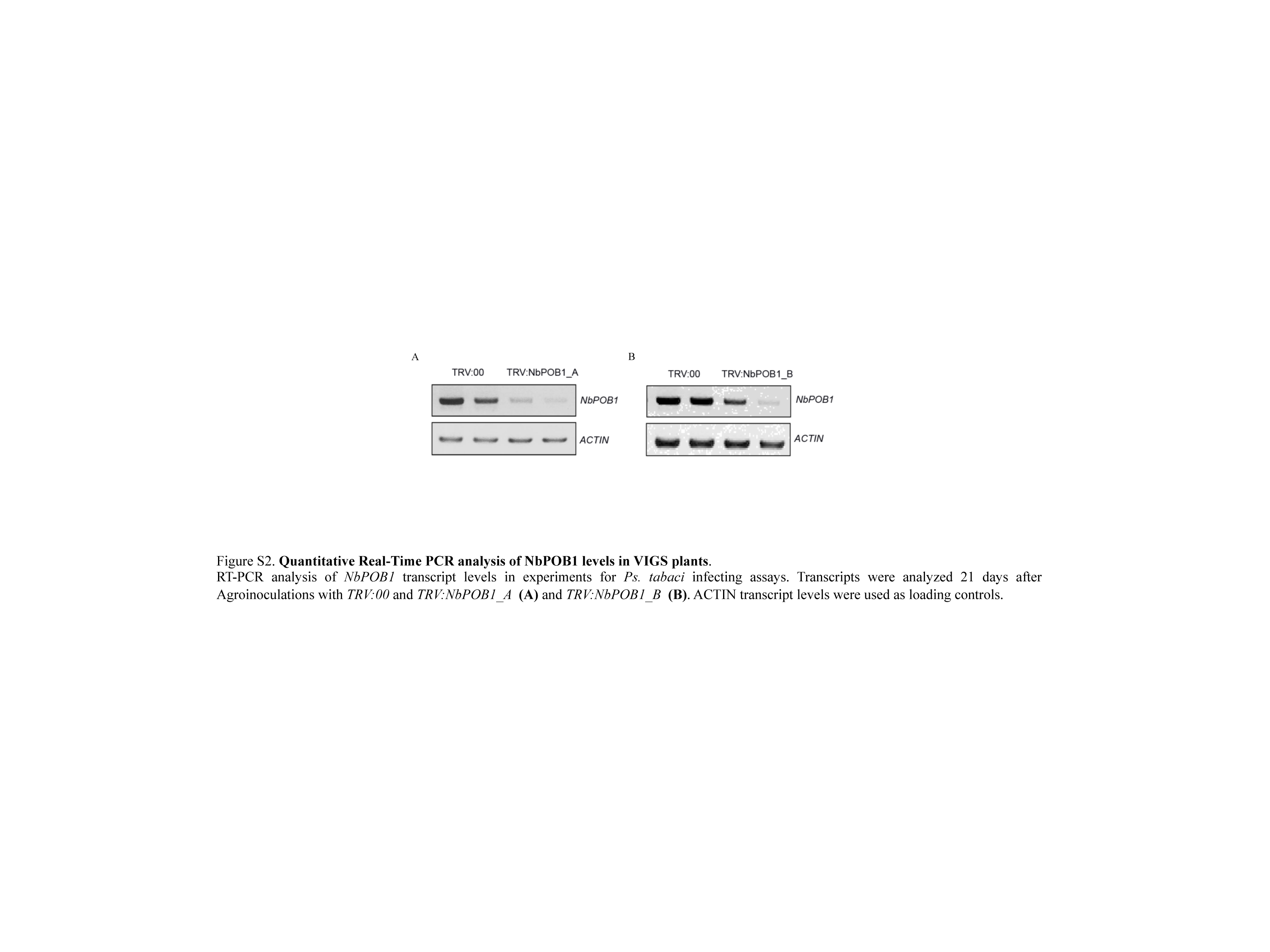

Supplement: S2 Fig — RT-PCR analysis of NbPOB1 transcript levels in experiments for Ps. tabaci infecting assays. Transcripts were analyzed 21 days after Agroinoculations with TRV:00 and TRV:NbPOB1_A (A) and TRV:NbPOB1_B (B). ACTIN transcript levels were used as loading controls. (TIF) [file pgen.1006540.s002.tif]

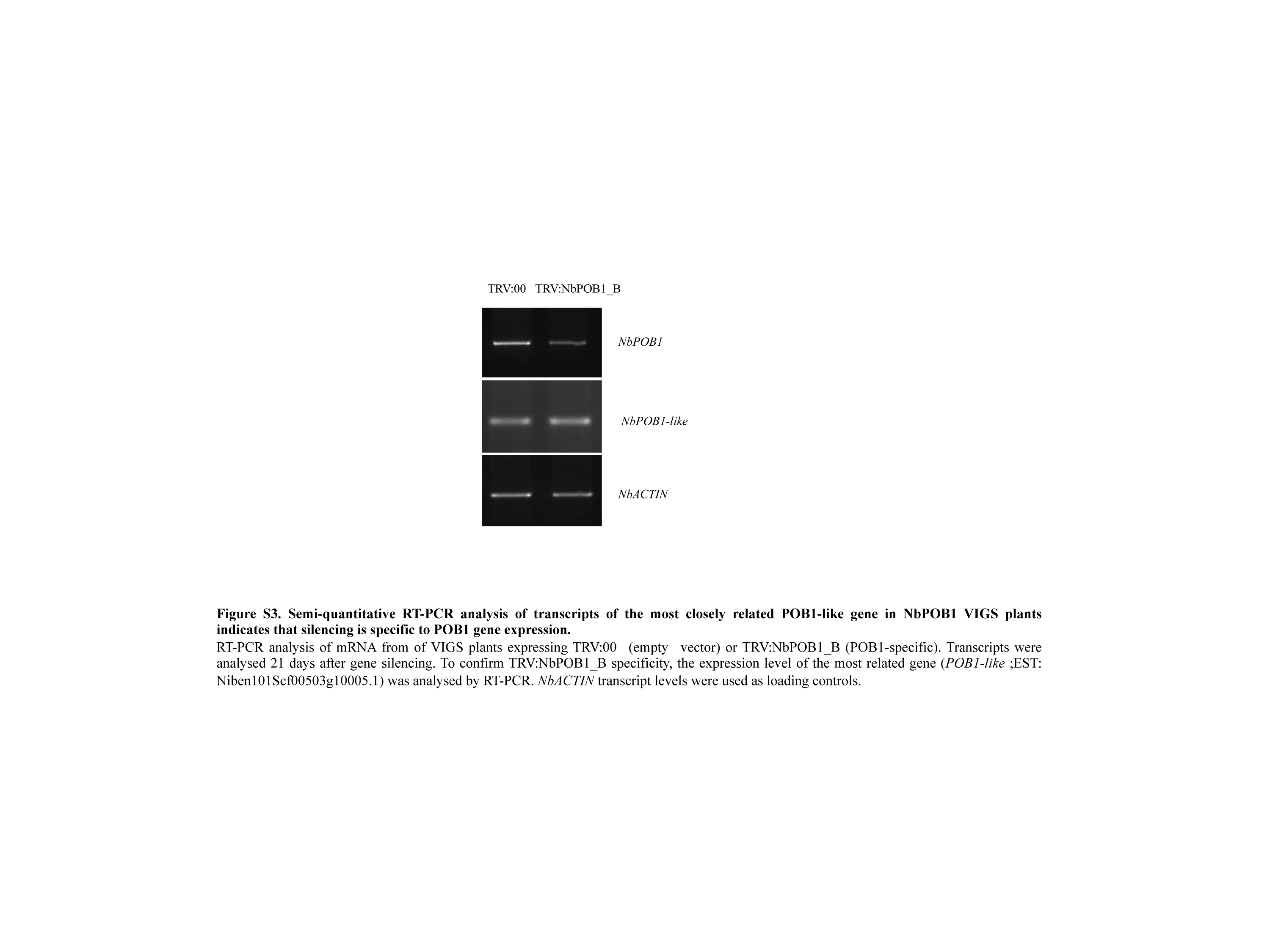

Supplement: S3 Fig — RT-PCR analysis of mRNA from of VIGs plants expressing TRV:00 (empty vector) or TRV:NbPOB1_B (POB1-specific). Transcripts were analysed 21 days after gene silencing. To confirm TRV:NbPOB1_B specificity, the expression level of the most related gene (POB1-like; EST: Niben101Scf00503g10005.1) was analysed by RT-PCR. NbACTIN transcript levels were used as loading controls. (TIF) [file pgen.1006540.s003.tif]

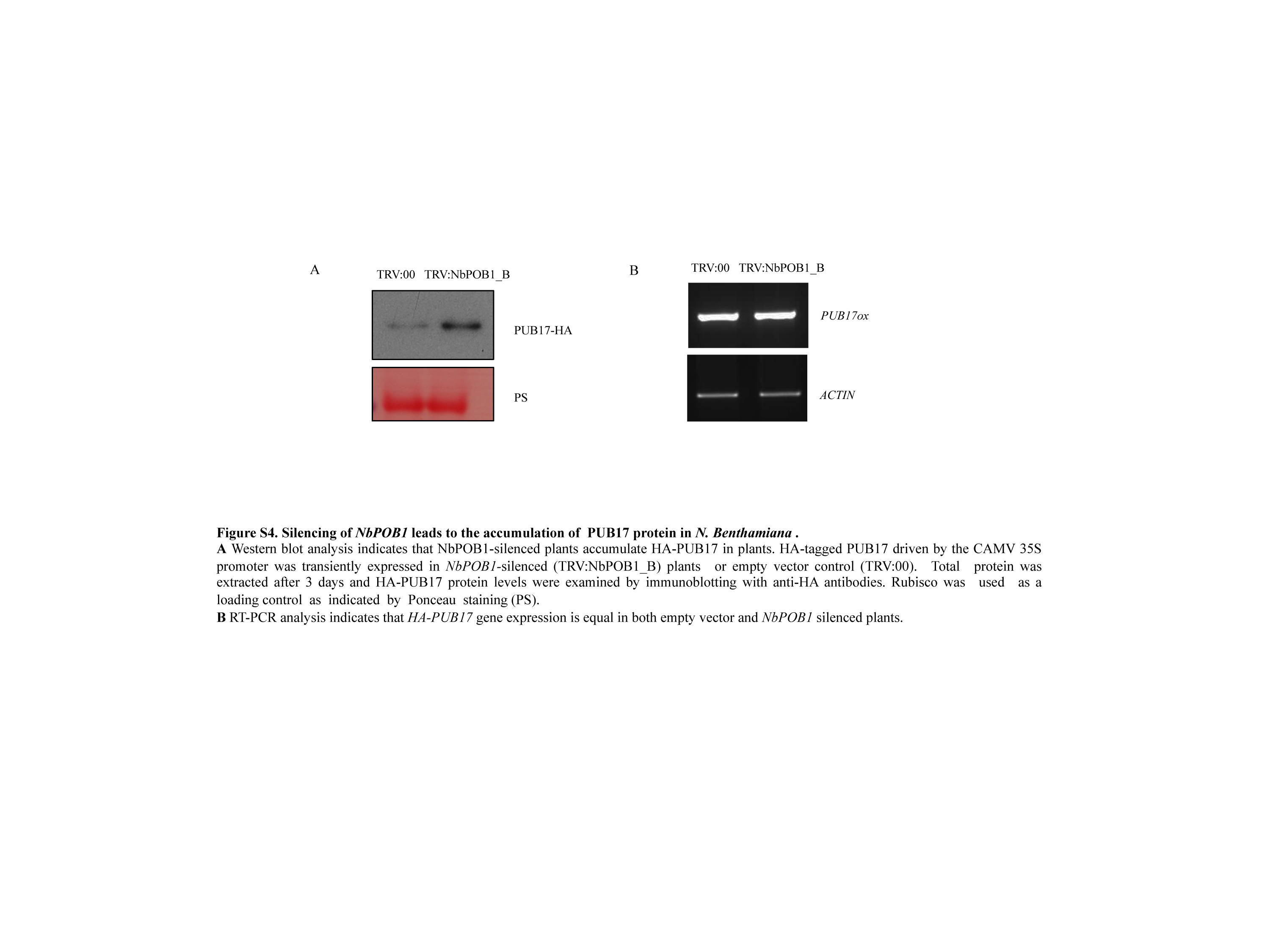

Supplement: S4 Fig — A. Western blot analysis indicates that NbPOB1-silenced plants accumulate HA-PUB17 in plants. HA-tagged PUB17 driven by the CAMV 35S promoter was transiently expressed in NbPOB1-silenced (TRV:NbPOB1_B) plants or empty vector control (TRV:00). Total protein was extracted after 3 days and HA-PUB17 protein levels were examined by immunoblotting with anti-HA antibodies. Rubisco was used as a loading control as indicated by Ponceau staining (PS). B. RT-PCR analysis indicates that HA-PUB17 gene expression is equal in both empty vector and NbPOB1 silenced plants. (TIF) [file pgen.1006540.s004.tif]

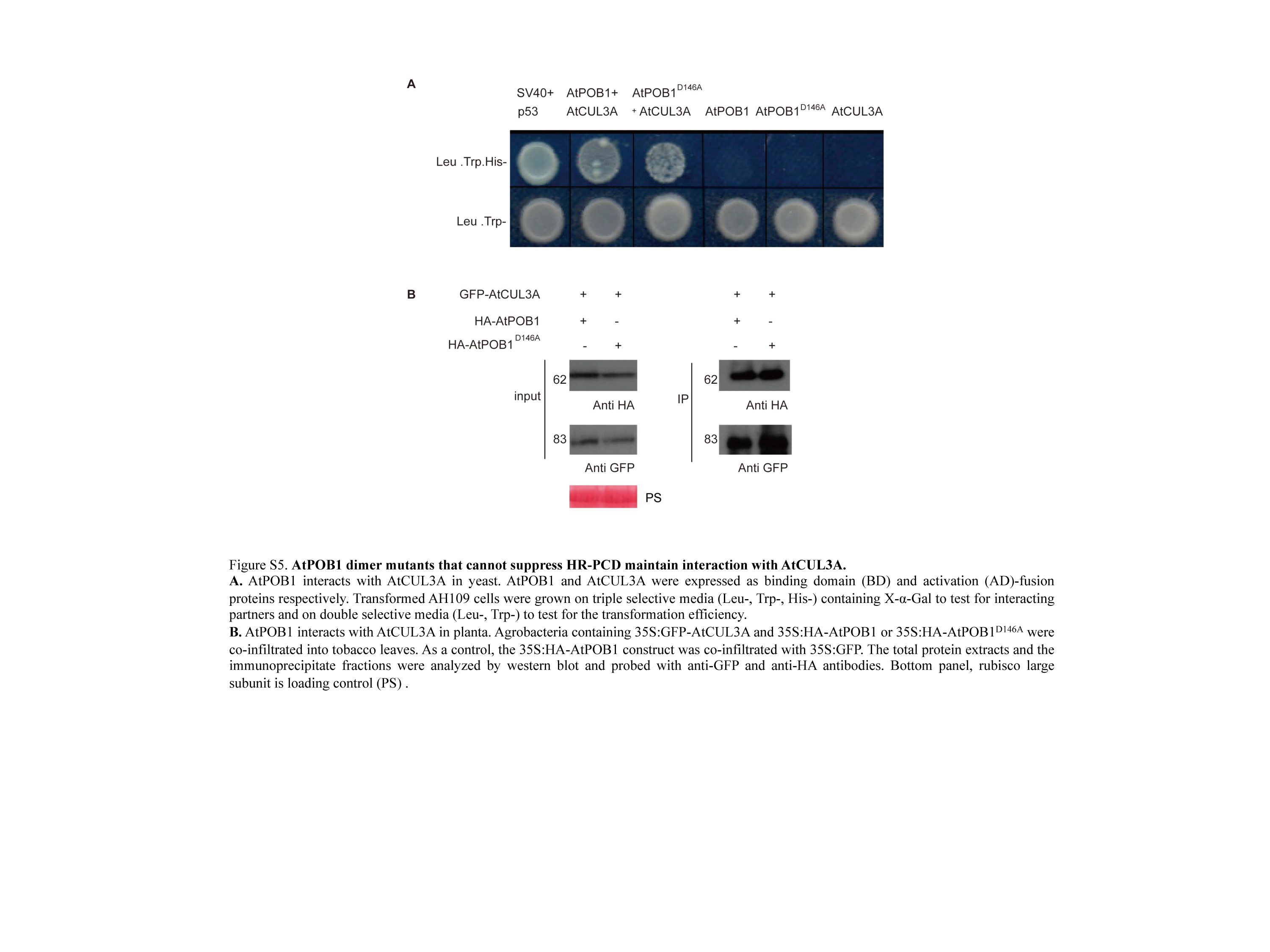

Supplement: S5 Fig — A. AtPOB1 interacts with AtCUL3A in yeast. AtPOB1 and AtCUL3A were expressed as binding domain (BD) and activation (AD)-fusion proteins respectively. Transformed AH109 cells were grown on triple selective media (Leu-, Trp-, His-) containing X-α-Gal to test for interacting partners and on double selective media (Leu-, Trp-) to test for the transformation efficiency. B. AtPOB1 interacts with AtCUL3A in planta. Agrobacteria containing 35S:GFP-AtCUL3A and 35S:HA-AtPOB1 or 35S:HA-AtPOB1D146A were co-infiltrated into tobacco leaves. As a control, the 35S:HA-AtPOB1 construct was co-infiltrated with 35S:GFP. The total protein extracts and the immunoprecipitate fractions were analyzed by western blot and probed with anti-GFP and anti-HA antibodies. Bottom panel, rubisco large subunit is loading control (PS). (TIF) [file pgen.1006540.s005.tif]
