# Supplementary material for: BTB-BACK Domain Protein POB1 Suppresses Immune Cell Death by Targeting Ubiquitin E3 ligase PUB17 for Degradation
Source: PLoS Genet. 2017 Jan 5;13(1):e1006540. doi: 10.1371/journal.pgen.1006540 (PMC5249250; doi:10.1371/journal.pgen.1006540)
Supplement: S2 Table — (DOCX) [file pgen.1006540.s007.docx]

| **Genes** | **Gene Constructs** | **vectors cloned into** | **experiments** |
| --- | --- | --- | --- |
| POB1 | GFP-AtPOB1 | pB7WGF2 | Localisation in *Nicotiana benthamiana* and transient expression assays |
|  | GFP-NtPOB1 | pB7WGF2 | transient expression assays |
|  | GFP-NbPOB1 | pB7WGF2 | transient expression assays |
|  | GFP-POB1^D146A^ | pB7WGF2 | transit expression assays |
|  | NES-GFP-POB1 | NES-pB7WGF2(NES-pB7WGF2:the NES signal sequence was inserted with annealed oligonucleotides into the unique *Spe*I site at the beginning of the GFP in the pB7WGF2 vector ) | Localisation in *Nicotiana benthamiana* and transient expression assays |
|  | YN-Myc-POB1 | PCL112 | transient expression assays for westerns and SplitYFP |
|  | BD-POB1 | pGBKT7 | Y2H |
|  | AD-POB1 | pGADT7 | Y2H |
|  | HA- POB1^D146A^ | pEG201 | transient expression assays for CO-IP |
|  | BD- POB1^D146A^ | pGBKT7 | Y2H |
|  | AD- POB1^D146A^ | pGADT7 | Y2H |
|  | HA-POB1 | pEG201 | transient expression assays for CO-IP |
|  | TRV2-POB1_A | TRV2: Tobacco Rattle Virus (TRV) vector (PDS) was cloned into TRV2 vector | Silencing NbPOB1 in *Nicotiana benthamiana* by VIGS |
|  | TRV2-POB1_B | TRV2: Tobacco Rattle Virus (TRV) vector (PDS) was cloned into TRV2 vector | Silencing NbPOB1 in *Nicotiana benthamiana* by VIGS |
|  | HG: NtPOB1 | pHELLSGATE12 | hairpin construct Silencing NtPOB1 in tobacco |
|  | HG: 00 | pTV00 |  |
| PUB17 | GFP-PUB17 | pB7WGF2 | transient expression assays for westerns and Co-IP |
|  | YC-HA-PUB17 | PCL113 | transient expression assays for westerns and SplitYFP |
|  | PUB17-HA | pEG201 | transient expression assays for westerns and Co-IP |
|  | BD-ARM |  | Y2H |
|  | BD-NtPUB17 | pGBKT7 | Y2H |
|  | AD-NtPUB17 | pGADT7 | Y2H |
| CUL3A | GFP-AtCUL3 | pB7WGF2 | transient expression assays for Co-IP |
|  | BD-AtCUL3 | pGBKT7 | Y2H |
|  | AD-AtCUL3 | pGADT7 | Y2H |
| GFP | EV | pB7WGF2 | The empty GFP control for transient expression assay in planta |
